# Dental caries experience, care index and restorative index in children with learning disabilities and children without learning disabilities; a systematic review and meta-analysis

**DOI:** 10.1186/s12903-019-0795-4

**Published:** 2019-07-15

**Authors:** Mark D. Robertson, Falk Schwendicke, Mariana Pinheiro de Araujo, John R. Radford, Jenny C. Harris, Scott McGregor, Nicola P. T. Innes

**Affiliations:** 1 School of Dentistry, University of Dundee, Park Place, Dundee, DD1 4HR UK; 2 Department of Operative and Preventive Dentistry, Charité - Universitätsmedizin Berlin, Berlin, Germany; 3 School of Dentistry, University of São Paulo, São Paulo, Brazil; 4 Community & Special Care Dentistry and Paediatric Dentistry, Charles Clifford Dental Services, Sheffield Teaching Hospitals NHS Foundation Trust, Sheffield, UK; 5 Dundee University Library, University of Dundee, Dundee, UK

**Keywords:** Children, Dental caries, Learning disability, Care index, Restorative index, Systematic review

## Abstract

**Background:**

Children with learning disabilities (CLD) have worse health outcomes than children with no learning disabilities (CNLD). This systematic review compared caries experience and met dental care need for CLD to CNLD using Decayed, Missing, Filled Permanent Teeth (DMFT) and decayed, missing/extracted, filled primary teeth (dmft/deft), care index (CI), and restorative index (RI) values.

**Methods:**

Without date or language restrictions four databases were searched for; cross-sectional studies comparing caries experience and CI/ RI in CLD matched to groups of CNLD. Screening and data extraction were carried out independently and in duplicate. Risk of bias was assessed using the Newcastle-Ottawa Scale. Meta-analyses were carried out (random effects model).

**Results:**

There were 25 articles with 3976 children (1 to 18 years old), from 18 countries, fitting the inclusion criteria. Children with; Down syndrome were investigated in 11 studies, autism in 8 and mixed learning disabilities in 6. The overall mean DMFT for CLD was 2.31 (standard deviation±1.97; range 0.22 to 7.2) and for CNLD was 2.51 (±2.14; 0.37 to 4.76). Using standardised mean difference (SMD), meta-analysis showed no evidence of a difference between CLD and CNLD (*n* = 16 studies) for caries experience (SMD = -0.43; 95%CI = -0.91 to 0.05). This was similar for sub-groups of children with autism (SMD = -0.28; 95%CI = 1.31 to 0.75) and mixed disabilities (SMD = 0.26; 95%CI = -0.94 to 1.47). However, for children with Down syndrome, caries experience was lower for CLD than CNLD (SMD = -0.73; 95%CI = -1.28 to − 0.18). For primary teeth, mean dmft/deft was 2.24 for CLD and 2.48 for CNLD (*n* = 8 studies). Meta-analyses showed no evidence of a difference between CLD and CNLD for caries experience across all disability groups (SMD = 0.41; 95% CI = -0.14 to 0.96), or in sub-groups: Down syndrome (SMD = 0.55; 95%CI- = − 0.40 to 1.52), autism (SMD = 0.43; 95%CI = -0.53 to 2.39) and mixed disabilities (SMD = -0.10; 95%CI = -0.34 to 0.14). The studies’ risk of bias were medium to high.

**Conclusion:**

There was no evidence of a difference in caries levels in primary or permanent dentitions for CLD and CNLD. This was similar for learning disability sub-groups, except for Down syndrome where dental caries levels in permanent teeth was lower. Data on met need for dental caries was inconclusive.

**Trial registration:**

The protocol was published in PROSPERO: CRD42017068964 (June 8th, 2017).

**Electronic supplementary material:**

The online version of this article (10.1186/s12903-019-0795-4) contains supplementary material, which is available to authorized users.

## Background

Disability affects approximately 1 million children in the UK, with around “8% of children aged 7-15 having specific educational needs associated with intellectual, developmental, communication, sensory or physical impairments in England alone” [1]. A learning disability can be defined as a reduced intellectual ability leading to challenges with everyday tasks and situations [2, 3]. It affects people for the duration of their lives and often requires significant support from carers to interact with others [4]. The disability’s impact on different aspects of life varies from person to person, depending on factors such as support from family, friends and carers but can significantly impact their healthcare access, ability to understand information, comply with instructions and cope with treatment. Not only do people with learning disabilities suffer from co-morbidities but they have worse health outcomes than their non-learning disabled counterparts in areas of health not related to their disability [5]. This is in part because they are more likely to have additional health problems [6] but can also be associated with suboptimal care from health care professionals and social services [5]. Differences in health status between people with learning disabilities and people without learning disabilities represents a genuine health inequality that is largely avoidable and entirely unjust [7].

Recent systematic reviews of those with intellectual disabilities have found conflicting evidence regarding their oral hygiene status. Oral hygiene was found to be poorer than in adults without learning disabilities [8]. In children with autism, oral hygiene has been found to be poorer and caries prevalence higher compared to the general population [9] and there is a question over whether children with Down syndrome have lower levels of caries as has been previously accepted [10]. Overall, caries experience of children with learning disabilities (CLD) compared to children without learning disabilities (CNLD) has not been clearly established, and the most recent review [11] did not limit the inclusion criteria to only children with learning disabilities. No previous reviews have attempted to look at the levels and type of dental care provided for managing carious teeth in CLD compared to CNLD. The care index and restorative index are measures of previous management of dental caries and build on the data from decayed, missing and filled teeth. As such, they give information on the delivery of dental services, inequalities in access, amount, and type of care that has been received (extraction or restorative). The well-established relationship between socio-economic status and dental caries [12], in addition to links between social deprivation and children with learning disabilities [13], suggests that they could suffer a greater burden of dental caries and unmet need where the disease exists.

The objective of this systematic review was to assess dental caries experience; and type and extent of dental care in children with learning disabilities in comparison to children without learning disabilities using DMFT/dmft, care index (CI), and restorative index (RI) values.

## Methods

The review was developed in accordance with Preferred Reporting Items for Systematic Review and Meta-Analysis Protocols (PRISMA-P) [14].

The review protocol was published in PROSPERO (June 8th, 2017; http://www.crd.york.ac.uk/PROSPERO/display_record.php?ID=CRD42017068964).

### Searches

There were no language or date restrictions imposed on the search. We recognised that terminology used to describe and group people with learning disabilities depends on cultural and historic contexts and evolves over time. Therefore we were inclusive of politically contested language while building search strategies.

The literature search strategies were developed using medical subject headings (MeSH) and text words related to dental caries in children and to disabilities. We searched four databases: MEDLINE (OVID interface, 1949 to June 2017); PubMed (1946 to June 2017); Scopus (Elsevier interface, 1996 to June 2017, 1823 to June 2017 without references); and Web of Science (1900 to June 2017) to find relevant literature using the following search strategy, which was designed for MEDLINE (Ovid) and afterwards revised for each database. The search was updated in December 2018:

(Child [MeSH] **OR** child* **OR** pediatric **OR** paediatric) **AND** (“pediatric dentistry”[MeSH] **OR** dentistry **OR** dental **OR** teeth **OR** tooth **OR** oral **OR** “oral health”[MeSH]) **AND** (“special needs” **OR** “special care” **OR** learning disabilities **OR** learning difficulties **OR** “learning disorders”[MeSH] **OR** “intellectual disability”[MeSH] **OR** mental retardation **OR** mentally retarded **OR** mongol* **OR** disab* **OR** autism **OR** autistic **OR** autistic spectrum **OR** “autistic spectrum disorder”[MeSH] **OR** Asperger syndrome **OR** “down syndrome”[MeSH] **OR** down* syndrome **OR** trisomy 21 **OR** dental care for disabled [MeSH]) **AND** (caries **OR** carious **OR** tooth decay **OR** lesions **OR** saliva* **OR** “salivation”[MeSH] **OR** “dental caries”[MeSH]) **AND** (DMF **OR** DMFS **OR** DMFT) **AND** (restorative index **OR** RI **OR** care index **OR** CI).

#### Inclusion criteria

##### Study design

Observational studies with data on caries experience collected through clinical examination of participants. Studies where CLD were compared to general population values were excluded.

##### Participants/ population

CLD (with or without physical disabilities) and a similar group of CNLD.

### Study selection

Two reviewers screened titles and abstracts, independently and in duplicate, against the inclusion and exclusion criteria, and, where there was disagreement, consensus was achieved through discussion with a third reviewer. Full texts were obtained for all titles which were thought to meet the inclusion criteria. Reviewers were not blinded to journal titles, study authors or institutions.

### Data extraction

Data extraction forms were piloted and feedback resulted in them being revised. The revised forms were used to collect data independently and in duplicate by two trained and calibrated reviewers.

#### Data extracted

- Study characteristics (title, reference, author(s) and year of publication);
  - ○ Methodology (Characteristics of data collection (e.g. data collectors, part of a regular epidemiological programme etc.); caries data collection system, and threshold for caries diagnosis (e.g. D2, D3 etc.);
- Year of data collection;
- Participant inclusion and exclusion criteria;
- Participant demographic information:
  - ○ Age groups were recorded. As the upper age limit for childhood varies geographically and between cultures, all groups were included up to the age of 19. Note was taken of the age group and range included in each study; and
  - ○ The type of disability was recorded as reported and decisions made around the appropriateness of grouping different disabilities together.
  - ○ Number of participants and whether or not a sample size was calculated.
  - ○ Study setting (country, region, national/ local/ international, and clinical setting).
- Outcome data for caries levels; and CI and RI.

The reviewers reached consensus through discussion where there was any discrepancy in data extraction.

### Data synthesis and meta-analysis

The mean DMFT/dmft for each study and the CI and RI were extracted or calculated from the data. The CI is a measure of the proportion of carious teeth that have been managed with restorations or by extraction and is defined as the number of restored teeth as a proportion of the total number of decayed (D), missing (M) and filled (F) teeth (CI=F/D + M + F). It provides an epidemiological measure of how much treatment has been provided to manage the disease. The RI is the proportion of carious teeth that have been filled as a proportion of the decayed and filled teeth (F/D + F). We planned to carry out subgroup analyses for primary/ permanent dentition, age and type of disability (after subgrouping depending on the data).

Included studies that reported DMFT and dmft values with variance estimates (*n* = 16) were included in the meta-analyses [15–30]. Generic-inverse variance method of meta-analysis was performed using Comprehensive Meta-Analysis 2.2.064 (Biostat, NJ, USA). Our outcome measure was the standardised mean difference (SMD), accounting for: (1) the difference in magnitude in caries experience across included age groups; and (2) the fact that some studies included deft, not dmft.

Two analyses on caries experience were performed, one for permanent teeth and one for primary teeth. Meta-analyses on RI/CI were not feasible given any kind of variance estimates were missing from all included studies.

Heterogeneity was assessed using Cochrane’s Q and I^2^-statistics [31]. Heterogeneity was always substantial (I2 > 95%), and so a random-effect models were used. Subgroup analyses was carried out for the three main groups of learning disabilities: Down syndrome, autism and mixed learning disabilities, and estimates compared across subgroups. Although the review protocol stated that the learning disabilities would be grouped depending on the literature, these particular subgroup analyses were exploratory as they had not been specifically planned a priori.

Publication bias was evaluated using Funnel plots as well as Egger’s regression intercept test [32]. We assumed asymmetric plots or significant test results (*p* < 0.05) to be an indication for publication bias.

### Risk of bias assessment (ROB)

An adapted Newcastle-Ottawa scale (NOS) [33] was used with a maximum score of ten points (5 for ‘Selection’, 2 for ‘Comparability’ and 3 for ‘Outcome’) spread across seven domains (Additional file 1: Appendix 1): sample representativeness; sample size; non-respondents; ascertainment of data on clinician’s decision to intervene at carious lesion thresholds; whether subjects in outcome groups are comparable, based on the study design or analysis, and confounding factors are controlled; assessment of the outcome; and statistical tests. This tool for assessing observational studies such as the ones included in this review has the advantage of allowing the quality of the studies to be compared with those in other reviews as it is commonly used. Scoring was undertaken by two reviewers, with a third reviewer resolving any disagreements. Studies were considered at low ROB when the overall scores were 9–10; medium ROB when scores were 6–8; and high ROB when they were 0–5.

## Results

### Searching/ screening results

**Fig. 1:**
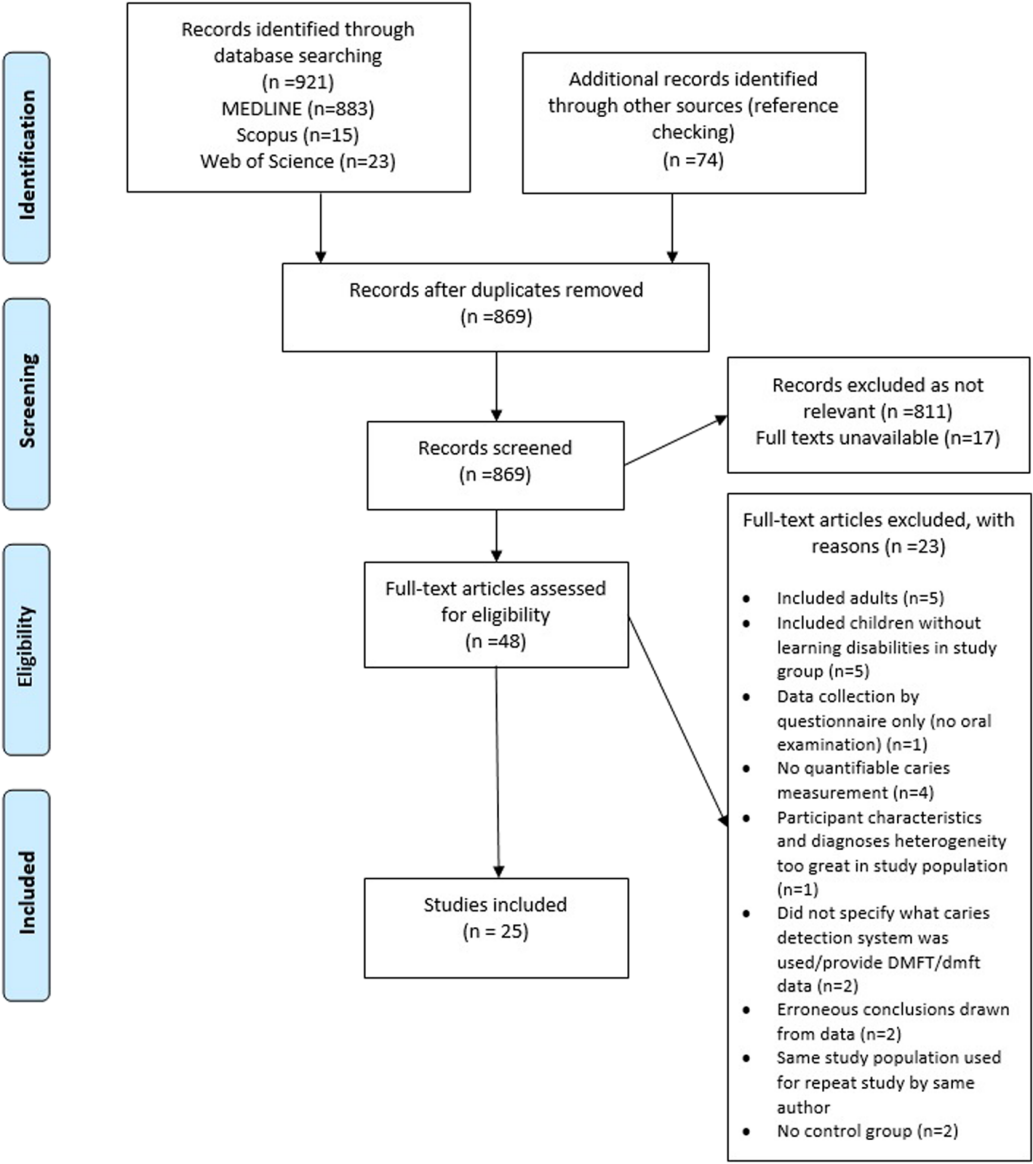
PRISMA [14] flow diagram of search results and screening of studies with reasons for exclusion and inclusion at full text screening

The searches resulted in 995 papers and 869 after de-duplication. Following title and abstract screening, 48 full papers were checked and 25 studies found to be eligible for inclusion (Fig. 1). Table 1 shows the excluded studies and the reasons why they were excluded.

**Table 1:** Full texts obtained and excluded with reason for exclusion (*n* = 23 studies)

Study	Reason for Exclusion
Areias et al., 2011 [34]	No quantifiable measure of DMFT/dmft - descriptive only.
Altun et al. 2010 [35]	Included children with physical disabilities as well as learning disabilities and data could not be separated.
Areias et al., 2012 [36]	This seems to be the same group or a repeat study dataset of Areias et al., 2013. This was not clear as it’s not detailed in the paper, but it included the same population of exactly the same age. DMFT and dmft were different but because of duplicate sampling, even if not the same dataset, we excluded.
Chadha et al., 2012 [37]	No comparison group
Bakarcic et al., 2009 [38]	Not all children in the study population have a learning disability
Fahlvik-Planefeldt et al., 2001 [39]	Index for caries recording/detection system not specified, no DMFT/dmft data
Fuertes-Gonzales et al., 2014 [40]	This study included adults (age range 2–37 years) and did not allow for extraction of age groups.
Fung et al., 2008 [41]	Data collection through questionnaire (no clinical examination carried out)
Lowe et al., 1985 [42]	This study included adults (age range 3–30 years) and did not allow for extraction of age groups.
Macho et al., 2013 [43]	This study included adults (age range 2–26 years) and did not allow for extraction of age groups.
Mattila et al., 2001 [44]	No learning disabled children in the study population.
Oredugba et al., 2007 [45]	This study included adults and did not allow for extraction of age groups for DMFT/dmft indices
Pollard et al., 1992 [46]	No learning disabled children in the study population.
Purohit et al., 2010 [47]	Not all children in the study population have a learning disability
Radha et al., 2016 [48]	Errors in study authors’ conclusions from dataset: Table 10 shows CLD to have a lower caries experience than CNLD group, however the discussion and conclusion sections state the opposite to be true.
Rai et al., 2012 [50]	Index for caries recording/detection system not specified, no DMFT/dmft data
Rekha et al. 2012 [51]	No DMFT/dmft data given, only caries prevalence
Ruiz et al., 2018 [52]	This study included adults (age range 4–20 years) and did not allow for extraction of age groups.
Sarnat et al., 2016 [53]	Index for caries recording/detection system not specified, no DMFT/dmft data
Shaw et al., 1985 [54]	Dataset includes disabled children from a very wide group also no consistency between children’s ages in the study group and control group.
Suhaib et al., 2017 [55]	No quantifiable measure of DMFT/dmft - descriptive only.
Subramanium et al., 2011 [56]	No comparison group
Weckwerth et al., 2016 [57]	Errors in study authors’ conclusions from dataset calculations; Table 1 demonstrates incorrect results for the CI calculations in the permanent dentition for both groups 1 and 2.

### Study characteristics

#### Study designs and sampling

Additional file 1: Appendix 2 shows the data tables with all extracted data. The studies were published between 1981 and 2017 with the number of papers published during the past 10 years (16 publications) greater than that number published during the previous three decades (9 publications). All 25 studies were comparative, cross-sectional in design although they varied in how the CLD and CNLD were sampled; in some studies the CNLD were siblings or other family members whereas in others they were sampled from wider representative populations. Where the CNLD were chosen from siblings or matched for age, gender etc. the studies were commonly referred to as case-control or by other terminologies with only five studies [17, 23, 28, 30, 58] correctly named as cross-sectional by the authors.

#### Study settings

The 25 studies were geographically spread across 18 different countries: one study from each of Argentina [20]; Croatia [59]; Egypt [22]; Finland [60]; Hong Kong [49]; Jordan [15]; Korea [61]; Libya [23]; Sweden [24]; UAE [26]; UK [62]; Yemen [16]; and Portugal [18]; two conducted in Israel [21, 63], Saudi Arabia [17, 64] and Turkey [58, 65], and three in each of Brazil [25, 27, 28] and India [19, 29, 30]. The clinical settings ranged from specialist Paediatric dental clinics to school classrooms under natural light (see Additional file 1: Appendix A, B and C).

#### Disability status and subgrouping

The disability status of the study participants ranged from single to combined impairments or multiple conditions. Nineteen studies had a main focus on one particular disability, with or without associated impairments. There were two distinct populations included in the studies; children with Downs syndrome (*n* = 11 studies) [15, 17, 18, 20, 21, 25, 27, 29, 61, 63, 65] and children with autism (*n* = 8 studies) [16, 19, 22, 23, 26, 30, 49, 58]. These accounted for over three quarters of the studies (19 out of 25). The remaining six studies focussed on groups of children with non-specific learning disabilities or considered study populations inclusive of a range of learning disability diagnoses [24, 28, 59, 60, 62, 64]. The studies were therefore grouped into those examining children with 1) Down syndrome, 2) autism, and 3) other mixed learning disabilities as per protocol for the subgroup analyses.

#### Non-participation and representativeness of participating groups

Across the studies, there tended to be few explicitly stated exclusion criteria. Where stated, children’s inability to cooperate, parental refusal, use of orthodontic appliances, systemic disease and absence of valid consent were cited (Additional file 1: Appendix 2). Four studies [26, 29, 61, 65] were more prescriptive in their exclusion criteria; systemic disease, previous disease, medication use, recent local infection and recent dental prophylaxis precluded participation. Eleven studies failed to report any exclusion criteria [17, 20, 21, 24, 27, 28, 58–60, 62, 63]. However, representativeness of the studies was acceptable with reasonable overall matching between control group and study group populations by age, gender and socio-economic status although the number of children in the matched group was sometimes inflated.

#### Caries assessment; examiners, recording indices and thresholds

There was no clear description of the person performing the clinical examination in three studies [18, 59, 62]. Data collection was by a single dentist in 17 studies [15, 16, 19, 20, 22, 24–27, 29, 30, 49, 58, 60, 61, 63, 65], two dentists in four studies [17, 21, 23, 64] and three dentists in one study [28] and methods varied for assessing and recording caries prevalence; the majority (*n* = 16 studies) used the World Health Organisation (WHO) caries detection system [15–17, 19–23, 26, 28, 29, 49, 58, 63, 65, 66], whilst alternative indices (DMF, NIDR, Rradike (1972) and Moller & Poulsen) were used in nine others [18, 24, 25, 27, 30, 59–62]. The threshold for caries recording was generally poorly reported and variable where stated. It ranged, for example, from “frank carious cavitation on any surface of any tooth” to “anything beyond a white spot lesion”.

#### Size of participant groups

Across the studies, the number of participants in the CLD groups ranged from 19 to 257 (mean = 73; sd = 47.3; media*n* = 62), and from 30 to 301 (mean = 86; sd = 64.6; median = 70) in the CNLD groups. (Tables 2 and 6). The number of children in the CLD groups was 1819 and in the CNLD was 2157, giving a total of 3976 children included in this review. The sizes of the participant groups by disability subgroup is shown in Tables 3 (Down syndrome), 4 (autism) and 5 (mixed disabilities).

**Table 2:** DMFT, Care index (CI) and Restorative index (RI) in CLD and CNLD (*n* = 25 studies; 20 studies with DMFT data and 16 with standard deviations reported). Studies where DMFT data couldn’t be used are shown in learning disability subgroup tables, with reasons why, but are not included in calculations data couldn’t be used are shown, with reasons why, but are not included in calculations. All data were calculated from the primary data reported in Additional file 1: Appendix 2

All learning disability groups (*n* = 20 studies with DMFT data and 16 with SD data)									
Study	Number of participants		Ages	DMFT data					
	CLD	CNLD		CLD DMFT (sd)	CNLD DMFT (sd)	CLD CI^a^	CNLD CI^a^	CLD RI^b^	CNLD RI^b^
Total for all 3 Groups	1819	2157	1–18						
Mean (sd)	72.76 (±47.31)	86.28 (±64.56)		2.31 (±1.97)	2.51 (±2.14)	0.12 (±0.12)	0.18 (±0.27)	0.15 (±0.14)	0.28 (±0.33)

^a^based on 8 studies

^b^based on 9 studies

**Table 3:** DMFT, Care index (CI) and Restorative index (RI) in CLD and CNLD (*n* = 25 studies; 20 studies with DMFT data and 16 with standard deviations reported). Studies where DMFT data couldn’t be used are shown in learning disability subgroup tables, with reasons why, but are not included in calculations data couldn’t be used are shown, with reasons why, but are not included in calculations. All data were calculated from the primary data reported in Additional file 1: Appendix 2

Down syndrome (permanent teeth; *n* = 11 studies)										
Study	Number of participants		Ages	DMFT data						
	CLD	CNLD		CLD DMFT (sd)	CNLD DMFT (sd)	CLD CI	CND CI	CLD RI	CNLD RI	Reason no DMFT data included
Al Habashneh 2012 [15]	103	103	12–16	3.32 (3.77)	4.59 (4.21)					
AlSarheed 2015 [17]	93	99	7–15	2.66 (3.09)	3.11 (2.58)	0.18	0.18	0.22	0.19	
Areias 2013 [18]	45	45	6–18	1.02 (2.42)	1.84 (3.13)	0.16	0.02	0.27	0.04	
Cogulu 2006 [65]	73	70	7–12	0.92	4.26					
Cornejo 1996 [20, 67]	86	86	10–13	1.30 (0.30)	1.70 (0.40)					
Davidovich 2010 [21]	70	32	1–9	3.37 (0.56)	5.90 (0.8)					
Hashizume 2017 [25]	61	52	6–14	0.36 (1.00)	0.40 (0.92)					
Lee 2004 [61]	19	41	8–17							DMFS only
Mathias 2011 [27]	69	69	1–7	2.20 (6.30)	3.40 (8.10)					
Stabholz 1991 [63]	32	30	8–13							DMFS only
Subramaniam 2014 [29]	34	34	7–12	1.68 (0.69)	1.84 (1.12)					
Subgroup Total	685	661								
Mean (sd)	62.27 (±26.97)	60.09 (±26.90)		1.87 (±1.08)	2.49 (±1.42)					

#### Participant ages

Participant age ranges were from 1 to 18 years-old (Tables 2, 3, 4, 5, 6, 7, 8 and 9), with two studies [67] reporting subgroups of dmf/DMF by age groups (Additional file 1: Appendix 2).

**Table 4:** DMFT, Care index (CI) and Restorative index (RI) in CLD and CNLD (n = 25 studies; 20 studies with DMFT data and 16 with standard deviations reported). Studies where DMFT data couldn’t be used are shown in learning disability subgroup tables, with reasons why, but are not included in calculations data couldn’t be used are shown, with reasons why, but are not included in calculations. All data were calculated from the primary data reported in Additional file 1: Appendix 2

Autism (permanent teeth; *n* = 8 studies)										
Study	Number of participants		Ages	DMFT data						
	CLD	CNLD		CLD DMFT (sd)	CNLD DMFT (sd)	CLD CI	CNLD CI	CLD RI	CNLD RI	Reason no DMFT data included
Al-Maweri 2014 [16]	42	84	5–16	2.00 (2.18)	1.27 (1.77)	0	0.02	0.04	0.03	
Bhandary 2017 [19]	30	30	6–12	0.37 (0.62)	0.37 (0.56)	0	0.19	0	0.35	
Du 2014 [49]	257	257	3–7			0.10	0.09	0.11	0.09	dmfs only
El Khatib 2014 [22]	100	100	3–13	3.40 (4.54)	3.50 (3.63)					
Fakroon 2014 [23]	50	50	3–14	0.22 (0.08)	1.15 (0.27)	0.1	0.06	0.1	0.07	
Jaber 2011 [26]	61	61	6–16	1.60 (0.64)	0.60 (0.29)					
Namal 2007 [58]	62	301	7–12	1.74	2.41	0.04	0.06	0.05	0.06	
Yashoda 2014 [30]	135	135	4–15	0.86 (1.22)	0.46 (1.06)					
Subgroup Total	737	1018								
Mean (sd)	92.13 (±74.74)	127.25 (±99.65)		1.10 (±0.69)	1.01 (±0.70)

**Table 5:** DMFT, Care index (CI) and Restorative index (RI) in CLD and CNLD (*n* = 25 studies; 20 studies with DMFT data and 16 with standard deviations reported). Studies where DMFT data couldn’t be used are shown in learning disability subgroup tables, with reasons why, but are not included in calculations data couldn’t be used are shown, with reasons why, but are not included in calculations. All data were calculated from the primary data reported in Additional file 1: Appendix 2

Mixed Learning Disability Groups (permanent teeth; *n* = 6 studies)										
Study	Number of participants		Ages	DMFT data						
	CLD	CNLD		CLD DMFT (sd)	CNLD DMFT (sd)	CLD CI	CND CI	CLD RI	CNLD RI	Reason no DMFT data included
Bakry 2012 [64, 66]	33	53	3–13					0.12	0.84	DFT only
Forsberg 1985 [24]	100	103	12–17	7.20 (6.10)	9.00 (4.00)					
Jokic 2007 [59]	80	80	3–17	6.39	4.76					
Moreira 2012 [28]	76	89	mean 8.9	5.20 (5.75)	1.50 (2.10)					
Palin 1982 [60]	58	58	9–10							no M or DMFS
Pope 1991 [62]	50	95	3–18	2.94	2.27	0.36	0.82	0.43	0.82	
Subgroup Total	397	478								
Mean (sd)	66.17 (±23.92)	79.67 (±20.24)		5.43 (±1.85)	5.20 (±2.79)

**Table 6:** dmft, Care index (CI) and Restorative index (RI) in CLD and CNLD (*n* = 25 studies; 9 studies with dmft and standard deviations reported). All learning disability groups. Studies where dmft data couldn’t be used are shown in learning disability subgroup tables, with reasons why, but are not included in calculations. All data were calculated from the primary data reported in Additional file 1: Appendix 2

All learning disability subgroups (primary teeth; *n* = 9 studies with dmft data)									
Study	Number of participants		Ages	dmft/deft data					
	CLD	CNLD		CLD dmft / deft (sd)	CNLD dmft / deft (sd)	CLD CI^a^ (sd)	CNLD CI^a^ (sd)	CLD RI^a^ (sd)	CNLD RI^a^ (sd)
Total for all 3 Groups	1819	2157							
Mean (sd)	72.76 (±47.31)	86.28 (±64.56)		2.34 (±1.37)	2.25 (±1.39)	0.15 (±0.14)	0.06 (0.06)	0.15 (±0.14)	0.04 (±0.01)

^a^ based on 2 studies

**Table 7:** dmft, Care index (CI) and Restorative index (RI) in CLD and CNLD (*n* = 25 studies; 9 studies with dmft and standard deviations reported). Down syndrome subgroup. Studies where dmft data couldn’t be used are shown in learning disability subgroup tables, with reasons why, but are not included in calculations. All data were calculated from the primary data reported in Additional file 1: Appendix 2

Down syndrome (primary teeth; *n* = 11 studies)										
Study	Number of participants		Ages	dmft/deft data						
	CLD	CNLD		CLD dmft / deft (sd)	CNLD dmft / deft (sd)	CLD CI	CNLD CI	CLD RI	CNLD RI	Reason no DMFT data included
Al Habashneh 2012 [15]	103	103	12–16							No dmft/deft data
AlSarheed 2015 [17]	93	99	7–15							No dmft/deft data
Areias 2013 [18]	45	45	6–18							No dmft/deft data
Cogulu 2006 [65]	73	70	7–12							No dmft/deft data
Cornejo 1996 [29, 49]	86	86	7–9	2.40 (0.60)	1.70 (0.30)					
Davidovich 2010 [21]	70	32	1–9							No dmft/deft data
Hashizume 2017 [25]	61	52	6–14	1.84 (3.67)	0.98 (1.39)					
Lee 2004 [61]	19	41	8–17							No dmft/deft data
Mathias 2011 [27]	69	69	1–7							No dmft/deft data
Stabholz 1991 [63]	32	30	8–13							No dmft/deft data
Subramaniam 2014 [29]	34	34	7–12	2.69 (1.62)	2.90 (1.60)					
Subgroup Total	685	661								
Mean (sd)	62.27 (±26.97)	60.09 (±26.90)		2.31 (±0.43)	1.86 (±0.97)

**Table 8:** dmft, Care index (CI) and Restorative index (RI) in CLD and CNLD (n = 25 studies; 9 studies with dmft and standard deviations reported). Autism subgroup. Studies where dmft data couldn’t be used are shown in learning disability subgroup tables, with reasons why, but are not included in calculations. All data were calculated from the primary data reported in Additional file 1: Appendix 2

Autism (primary teeth; *n* = 8 studies)										
Study	Number of participants		Ages	dmft/deft data						
	CLD	CNLD		CLD dmft / deft (sd)	CNLD dmft / deft (sd)	CLD CI	CNLD CI	CLD RI	CNLD RI	Reason no DMFT data included
Al-Maweri 2014 [16]	42	84	5–16	5.23 (2.34)	4.06 (2.98)	0.05	0.10			
Bhandary 2017 [19]	30	30	6–12							No dmft/deft data
Du 2014 [49]	257	257	3–7							No dmft/deft data
El Khatib 2014 [22]	100	100	3–13	3.53 (4.57)	3.56 (3.86)					
Fakroon 2014 [23]	50	50	3–14	1.13 (1.84)	2.85 (3.32)	0.25	0.02	0.25	0.03	
Jaber 2011 [26]	61	61	6–16	0.80 (0.20)	0.30 (0.30)					
Namal 2007 [58]	62	301	7–12							No dmft/deft data
Yashoda 2014 [30]	135	135	4–15	0.40 (2.48)	0.59 (1.28)					
Subgroup Total	737	1018								
Mean (sd)	92.13 (±74.74)	127.25 (±99.65)		2.42 (±1.90)	2.27 (±1.73)

**Table 9:** dmft, Care index (CI) and Restorative index (RI) in CLD and CNLD (*n* = 25 studies; 9 studies with dmft and standard deviations reported). Mixed learning disabilities subgroup. Studies where dmft data couldn’t be used are shown in learning disability subgroup tables, with reasons why, but are not included in calculations. All data were calculated from the primary data reported in Additional file 1: Appendix 2

Mixed Learning Disability Groups (primary teeth; *n* = 6 studies)										
Study	Number of participants		Ages	dmft/deft data						
	CLD	CNLD		CLD dmft / deft (sd)	CNLD dmft / deft (sd)	CLD CI	CNLD CI	CLD RI	CNLD RI	Reason no DMFT data included
Bakry 2012 [64, 66]	33	53	3–13					0.05	0.04	No dmft/deft data
Forsberg 1985 [24]	100	103	3–11	2.00 (2.90)	3.30 (2.80)					
Jokic 2007 [59]	80	80	3–17							No dmft/deft data
Moreina 2012 [28]	76	89	mean 8.9							No dmft/deft data
Palin 1982 [60]	58	58	9–10							No dmft/deft data
Pope 1991 [62]	50	95	3–18							No dmft/deft data
Subgroup Total	397	478								
Mean (sd)	66.17 (±23.92)	79.67 (±20.24)		2.00	3.30					

### Caries experience in the permanent teeth for CLD compared to CNLD (DMFT)

Of the 25 studies, 20 reported DMFT, two DMFS [38, 61, 63], one DFS [60], one DFT [64] and one reported on primary teeth only [49] (Additional file 1: Appendix 2 and Tables 2, 3, 4, 5, 6, 7, 8 and 9). For the 20 studies reporting DMFT, eleven included children with Down syndrome [15, 17, 18, 20, 21, 25, 27, 29, 61, 63, 65], eight with autism [16, 19, 22, 23, 26, 30, 49, 58] and six mixed disability groups [24, 28, 59, 60, 62, 64]. The overall mean DMFT for the CLD was 2.31 (sd ± 1.97; range 0.22 to 7.2) and for CNLD was 2.51 (sd ±2.14; range 0.37 to 4.76). The mean DMFT value for the subgroup of children with Down syndrome was 1.87 (sd ±1.08; range 0.36 to 3.37) and for the comparison group higher at 2.49 (sd ±1.42; range 0.4 to 4.59) (Table 3). For children with autism (Table 4), the mean DMFT was 1.10 (sd ±0.69; range 0.22 to 2.00) and 1.01 (sd ±0.70; range 0.37 to 2.41) in the comparison groups. In the four studies with children who had mixed learning disabilities (Table 5) [24, 28, 59, 62] the mean DMFT for CLD was 5.43 (sd ±1.85; range 2.94 to 7.2) and 5.20 (sd ±2.79; range 2.27 to 9.0) for CNLD.

**Fig. 2:**
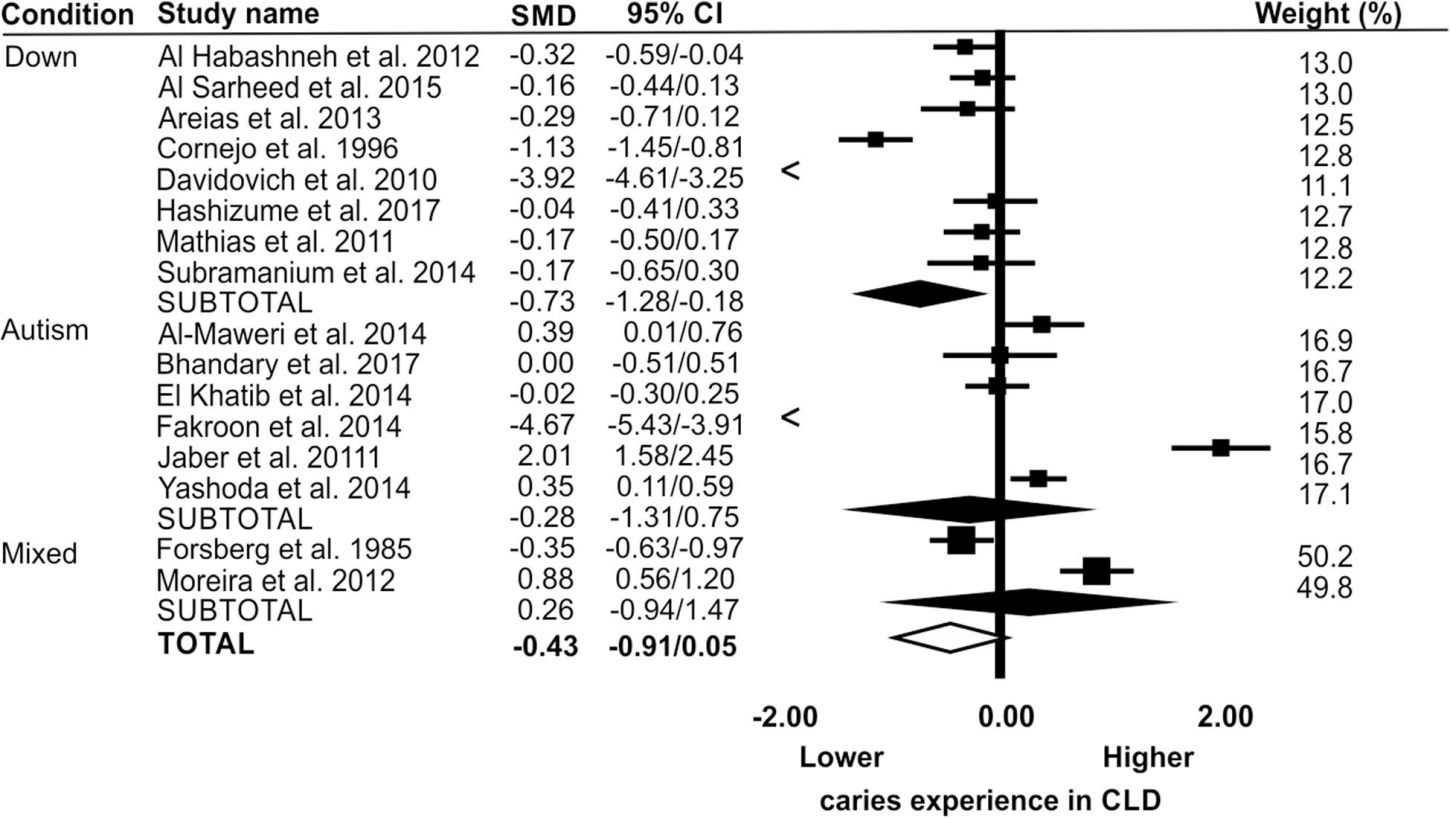
Caries experience of CLD compared to CNLD for permanent teeth (DMFT) presented as standard mean differences (SMD) and 95% confidence interval (CI). Full diamonds are subtotals for each of the three learning disability groups (Down syndrome, autism and mixed learning disability) and the open diamond indicates the overall difference in caries experience between the three groups

There were 16 papers that reported DMFT, where means and variance data were available [15–30]. Heterogeneity in meta-analysis was high (I^2^ > 95%) therefore a random effects meta-analysis was performed. Meta-analysis found no evidence of a difference between the CLD and CNLD for caries experience in the permanent dentition across all disability groups (SMD = -0.43; 95% CI = -0.91 to 0.05), for children with autism (SMD = -0.28; 95% CI = 1.31 to 0.75) or mixed disabilities (SMD = 0.26; 95% CI = -0.94 to 1.47). However, for children with Down syndrome there was a lower caries experience in the CLD compared to the CNLD (SMD = -0.73; 95% CI = -1.28 to − 0.18) (Fig. 2). [15–17, 19, 20, 25]

### Caries experience in the primary dentition for CLD compared to CNLD (dmft)

For primary teeth, dmft/deft values were reported in nine studies [16, 20, 22, 23, 25, 26, 30] [24, 29]; with a mean dmft/deft of 2.24 for CLD and 2.48 for CNLD. Two studies only reported dft values [59, 66] (Additional file 1: Appendix 2).

For the three studies with children who had Down syndrome [20, 25, 29], the mean dmft/deft was 2.31 (sd ±0.43; range 1.84 to 2.69) for CLD and 1.86 (sd ±0.97; range 0.98 to 2.90) for CNLD with two of the three studies finding the dmft to be higher for the CLD,

There were five studies on autism [16, 22, 23, 26, 30] . The mean dmft/deft was 2.42 (sd ±1.90; range 0.80 to 5.23) for CLD and 2.27 (sd ±1.73; range 0.30 to 4.06) for CNLD. Three studies reported children with autism to have a higher caries experience than children without autism [16, 26, 30].

The study of deft for children with other disabilities [24] found a deft of 2.00 for CLD and 3.30 for CNLD.

**Fig. 3:**
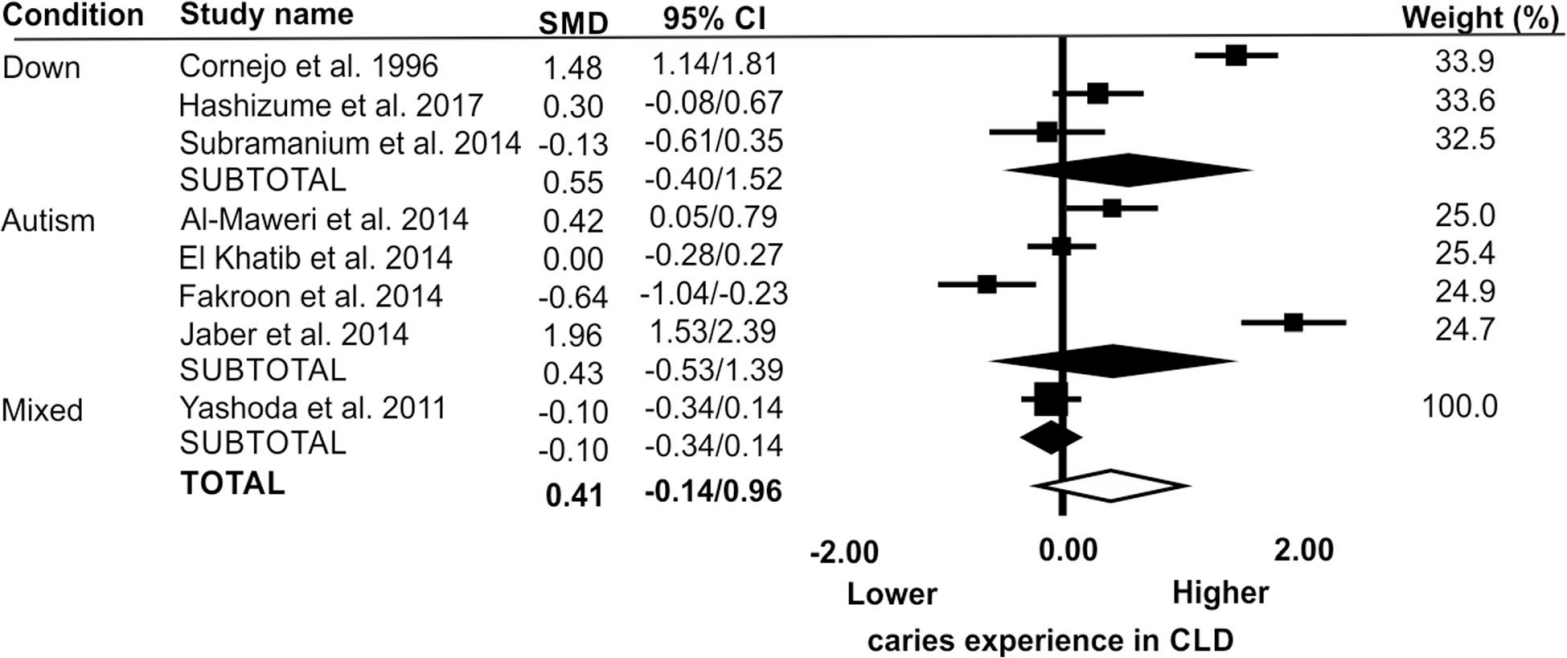
Caries experience of CLD compared to CNLD for primary teeth (dmft) presented as standard mean differences (SMD) and 95% confidence interval (CI). Full diamonds are subtotals for each of the three learning disability groups (Down syndrome, autism and mixed learning disability) and the open diamond indicates the overall level of caries experience between the two groups

There were eight papers [16, 20, 22, 23, 25, 26, 29, 30] that reported means and variance for dmft (Fig. 3). Meta-analyses found no evidence of a difference between the CLD and CNLD for caries experience across all disability groups (SMD = 0.41; 95% CI = -0.14 to 0.96), for children with Down syndrome (SMD = 0.55; 95% CI = -0.40 to 1.52), autism (SMD = 0.43; 95% CI = -0.53 to 2.39) or mixed disabilities (SMD = -0.10; 95% CI = -0.34 to 0.14).

#### Care, and restorative indices (permanent teeth)

There were eight studies [16–19, 23, 49, 58, 62] that stated the CI and RI or had data (decayed, missing and filled teeth values) that allowed calculation of both and one stated only the RI [64]. In five of eight studies, the CI for CLD was less than or equal to CNLD [16, 17, 19, 58, 62]. The RI for the permanent dentition was available for nine studies [16–19, 23, 26, 49, 58, 62, 64]. The RI for CLD was less than or equal to CNLD in four [19, 58, 62, 66] of the nine studies where it was available [16–19, 23, 26, 49, 58, 62, 64]. Meta-analysis was not possible on the data as there were no variance data given for any studies.

#### Care index and restorative index (primary teeth)

Two studies, both on children with autism, allowed calculation of the CI in the primary dentition [16, 23]. One [16] found it to be higher in CNLD, whilst the other [23] found it to be higher in CLD. Similarly, the two studies where calculation of the RI was possible reported conflicting findings; Bakry et al. (2012) found the RI to be higher in CLD whilst Fakroon et al. [23] found it to be higher in CNLD.

#### Narrative reports of unmet dental need

An additional six papers [26, 67–69] gave narrative reports on levels of unmet dental need and common treatment modalities (Table 10). Five of the six studies [26, 67, 69] noted a greater unmet treatment need in the CLD population although there were no numerical data to verify these statements.

**Table 10:** Included studies with descriptions of levels of unmet dental need where these were reported narratively but without supporting numerical data

Study	Disability for CLD group	Relative level of unmet dental need in CLD compared with CNLD	Narrative text on dental care provided for caries
Cornejo 1996 [29, 49]	Down	Higher for CLD	DS children receive less treatment of the deciduous dentition - this may be due to the delay in eruption of the teeth when examined alongside non-DS children of a similar age.
El Khatib 2014 [22]	Autism	Higher for CLD	In the primary dentition, children with ASD had more untreated caries. In the mixed stage, ASD children with ASD had less filled teeth than children without ASD.
Jaber 2011 [26]	Autism	Higher for CLD	Autistic children receive 60% less treatment.
Palin 1982 [60]	Range of conditions	Higher for CLD	In comparison with the healthy, the retarded children are not given enough dental care with respect to their treatment need.
Stabholz 1991 [63]	Down	Higher for CLD	Authors hypothesise that because treating those with DS and MR (institutionalised) is more expensive, more complex and requires more specialised personnel, only a small proportion of their needs are met.
Forsberg 1985 [24]	Range of conditions	Lower for CLD	The severely mentally retarded children had been offered dental care to the same extent as healthy children

### Quality assessment

Intra-study ROB ranged from 2 to 9, with a mean score of 5.2 showing a generally medium to high risk of bias across the studies. One study only scored two points out of the 10 possible [59]. Each study could be awarded a maximum score of 10 points across three domains. The mean scores across each domain were: 1.6 out of a possible score of 2 points for ‘comparability’; 1.9 out of 5 for ‘selection’; and 1.6 out of 3 for ‘outcome’. Five studies described the sample size calculations [19, 28, 70].

Of the 25 studies in the systematic review, the breakdown of quality assessment scoring, as per the Newcastle-Ottawa Scale (NOS) was as follows (see Additional file 1: Appendices 3A, 3B and 3C for full details):

- High quality (scoring 9–10 out of 10 across the three domains) *n* = 1 [23];
- medium quality (scoring 6–8 out of 10 across the three domains) *n* = 9 [15–17, 22, 29, 49, 60, 65, 66]; and
- low quality (scoring 0–5 out of 10 across the three domains) *n* = 15 [18–21, 24–28, 30, 58, 59, 61–63].

### Assessment of publication bias

**Fig. 4:**
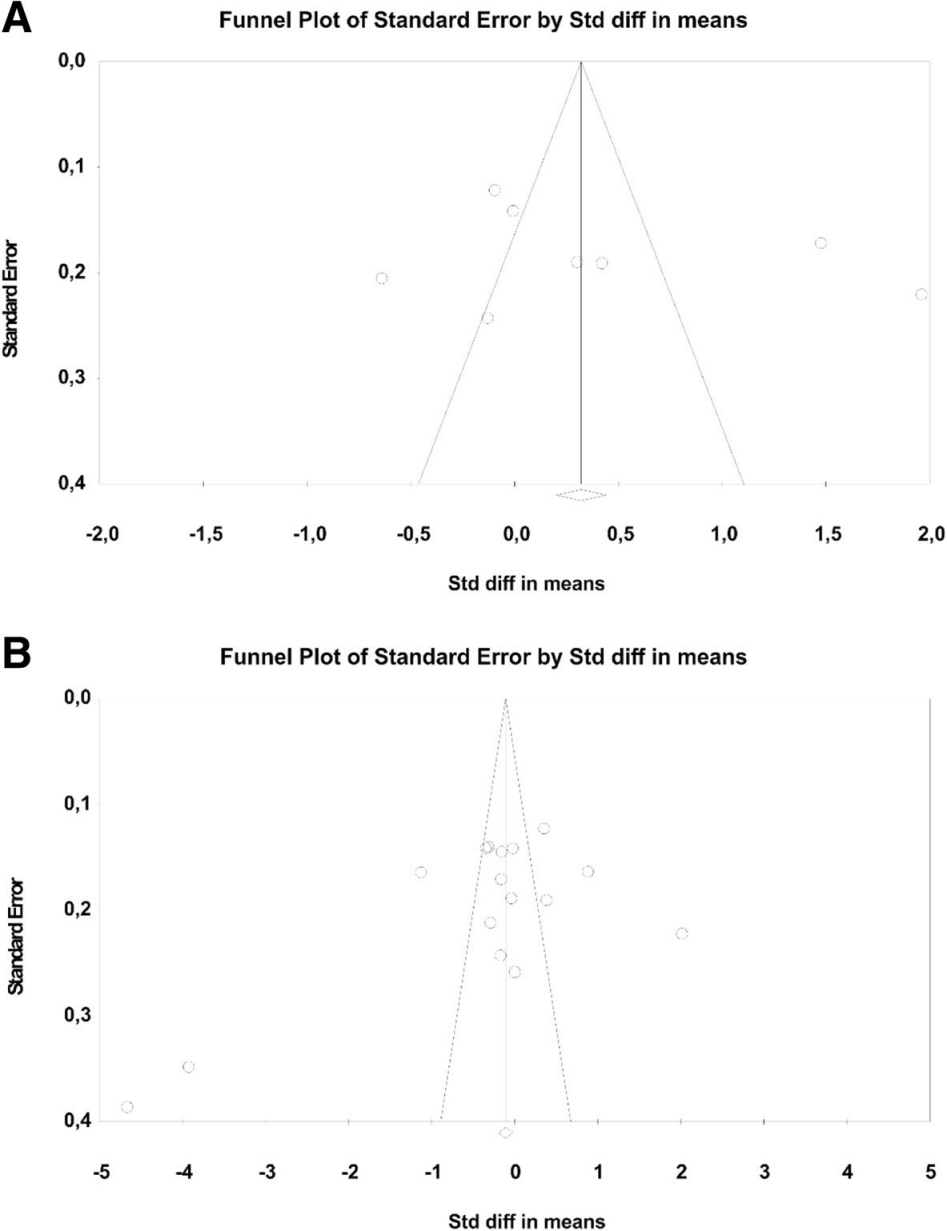
**a** Funnel plot to investigate publication bias in permanent dentition. **b** Funnel plot to investigate publication bias in primary dentition

There was no evidence of publication bias from the symmetry of the Funnel plots or Egger’s regression intercept test (Fig. 4a and b).

## Discussion

There is a lack of evidence around the impact that disability has on oral health and oral healthcare experience for children [10, 11]. This systematic review found 25 comparative, cross-sectional studies set in 18 countries including 3976 children (CLD 1819; CNLD 2157). This seems to be an area of growing interest, with almost twice as many publications in the last 10 years than in the previous 30 years. None of the recent three systematic reviews [10, 11] in this area seemed to find all of the papers we included. However, we involved an information technologist to help find the correct terms and papers and it was noted that research in this area is very poorly “tagged” in electronic literature databases with a lack of standardised keywords and therefore is difficult to find.

The two main groups of children with learning disabilities that were investigated were those with Down syndrome and those with autism, comprising three quarters of the sample of studies. The other group that seems to be of interest are children with cerebral palsy. However, reporting characteristics of children with cerebral palsy in these studies were poor, with very few specifying whether the children had any learning disability. As this is not one of the defining features of cerebral palsy [71] we only included studies where there was explicit information on the inclusion of children with learning disabilities.

Meta-analyses between studies allows pooled estimates using weighted averages for different studies. This method of data aggregation gives greater statistical power, more precise point estimates and the ability to look for patterns in results, for example through sub-group analyses. However, its correct use relies partly on appropriate assumptions being made about the similarity of studies. There were a number differences between the studies encompassed in this review including: comparison groups (some studies chose siblings of similar ages, others matched the CLD group with children from the general population of same gender and age); age groups included; type of learning disability; and the carious lesion diagnostic threshold (although this was not consistently well reported). However, we considered that there was enough homogeneity across the samples and similarities between the groups to be able to carry out meta-analyses.

For permanent teeth, meta-analysis included 16 out of the 20 studies where DMFT was available. It showed no evidence of a difference between CLD and CNLD for caries experience (SMD:-0.43; 95% CI:-0.91 to 0.05) or for subgroup analyses by disability for children with autism (SMD = -0.28; 95% CI = 1.31 to 0.75) and mixed disabilities (SMD = 0.26; 95% CI = -0.94 to 1.47). However, for children with Down syndrome caries experience was lower for CLD than CNLD (SMD = -0.73; 95% CI = -1.28 to − 0.18).

For primary teeth, meta-analysis could be carried out, with eight studies and showed no evidence of a difference between CLD and CNLD for caries experience across disability groups (SMD = 0.41; 95% CI = -0.14 to 0.96) or in sub-group analyses by disability group: Down syndrome (SMD = 0.55; 95% CI = -0.40 to 1.52), autism (SMD = 0.43; 95% CI = -0.53 to 2.39) and mixed disabilities (SMD = -0.10; 95% CI = -0.34 to 0.14).

There is some disagreement in the literature around whether those with Down syndrome experience less caries than the general population however a recent systematic review [10] found no evidence that people with Down syndrome have a lower experience of dental caries than non-syndromic individuals. Key features of Down syndrome such as hypodontia and microdontia have been suggested as possible factors contributing to this (by making proximal surfaces accessible to saliva and toothpaste). However, there has also been quite extensive investigation of biological factors that convey some protection such as salivary secretory IgA [61, 65]. One of the complicating factors in trying to interpret data for Down syndrome children and caries experience is the delay in tooth eruption which might also convey some protection. In one study where the delay in tooth eruption was taken into consideration, the caries experience of those participants with Down syndrome compared to non-syndromic individuals became not significant [72]. This suggests that studies investigating caries experience in people with Down syndrome should take into consideration timing of tooth eruption and not just participant ages.

As well as looking at levels of dental caries, our aspiration with this paper had been to look at levels and type of dental care provision using the care- and restorative- indices [73]. However, although there were mean values for eight studies for the CI and in nine studies for the RI, there were no standard deviations to show variance around the means for any of the studies. This meant that no meta-analyses could be carried out for permanent teeth. The mean CI (CLD: 0.12 ± 0.12; CNLD: 0.18 ± 0.27) and RI (CLD: 0.18 ± 0.27; CNLD: 0.28 ± 0.33) values showed a greater difference between the CI and RI for the CNLD than CLD (although it should be noted that the standard deviations are large). Only three studies provided CI and/or RI data for primary teeth and these showed mixed results regarding the quantity of care provided, and the nature of the interventions therefore no conclusions could be drawn [16, 23, 64].

**Fig. 5:**
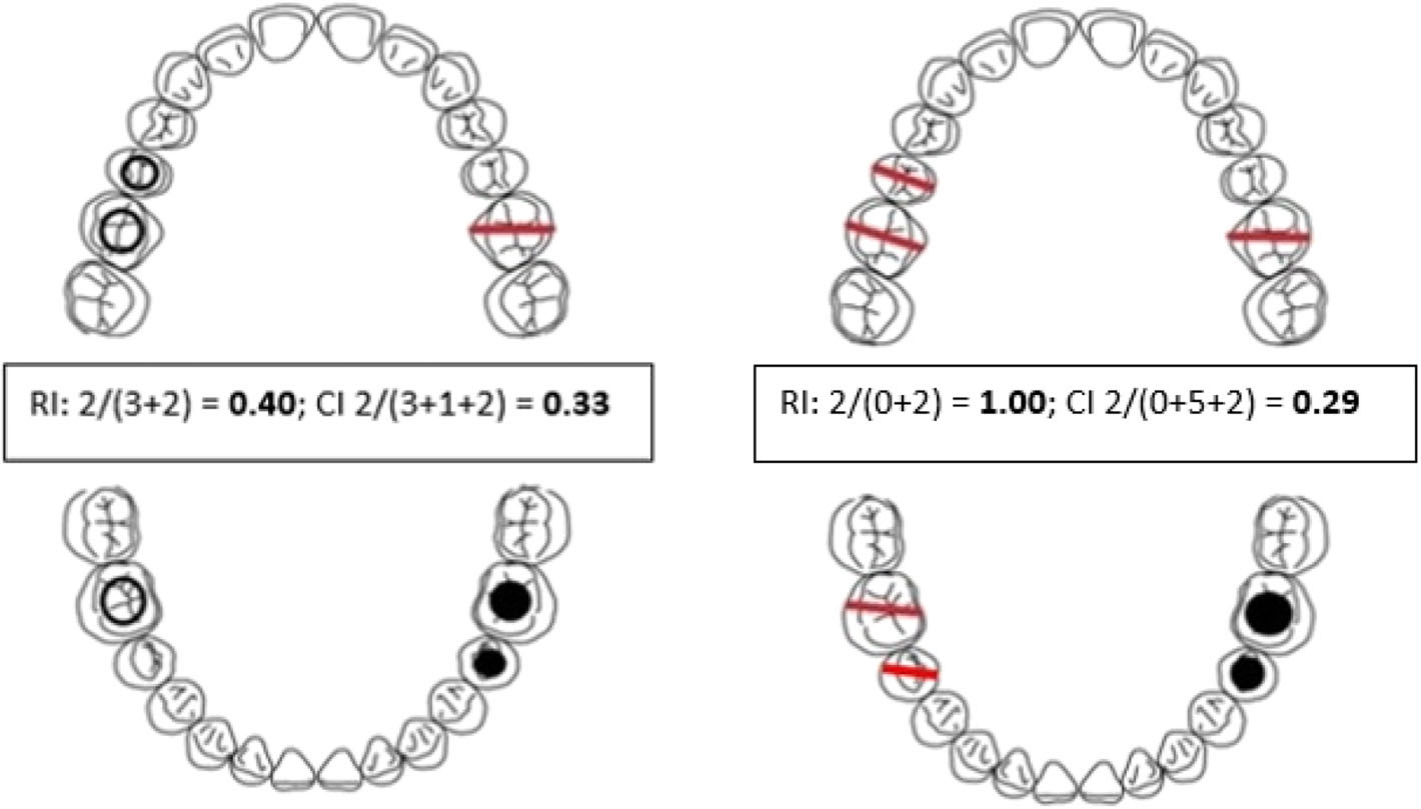
Diagrammatic representation of differences in CI and RI. There are two individuals with similar caries experience, the CI is similar for both (0.33 and 0.29) but the RI is only 0.4 for the one who has had less restorative care and reaches 1.00 for the one who has had additional carious teeth extracted rather than left carious. This shows that the greater the difference between the CI and RI, the more extraction based treatment has been used rather than restorative based treatment to manage carious teeth

Missing teeth are not included in the RI but are included in the CI formula; the higher the number of missing/ extracted teeth compared to restored teeth, the greater the difference between the CI and the RI as illustrated in Fig. 5.

For the permanent dentition the CI and RI values for CNLD were similar, whereas for CLD the RI tended to be higher than the CI (Table 2). This means that there are more teeth being extracted than restored in the CLD to manage their carious teeth.

This was corroborated by five of the six papers that narratively (but with no quantitative data) reported on the levels and types of dental care for CLD (Table 10). This data was not in the original protocol or inclusion criteria and was extracted *post-hoc*. Whilst this means that some studies with narrative information on levels and types of dental care provision may not have been included, this kind of data is likely to only have been found in papers that quantitatively analysed caries rates and this was an inclusion criterion. This means that there was a low risk of studies including this narrative information not being found during the search and included. Whether data is quantitative or narrative, there are four areas to consider in making judgements on data synthesis: 1) the direction of the effect 2) the size of any effect 3) consistency across studies and 4) strength of evidence [74]. There was sparse data available from the included studies quantitatively through the CI and RI (limited by the lack of variance data) and narratively through the descriptions of amount and type of dental care provided in CLD. However, the consistency of data – that there seemed to be a tendency for extraction based care over restorative based care - indicates a need for further research on this topic to ensure service is being tailored to those who need it. Poor reporting of research, making it not possible to use collected data in secondary analyses, is indicative of a much wider problems of waste in research [75]. Overall, the included studies were not of high quality (from a risk of bias perspective). In addition, the reported data was simply not included by the authors in many cases. This was seen in the reporting of variance around DMFT/dmft values, and in authors’ frequent failure to report underlying data on decayed, missing and filled teeth values which precluded calculation of CI and RI indices. Finally, even when this was reported, there were no variance values in any of the studies. Improved reporting and including detailed datasets would significantly reduce this problem and improve the quality of subsequent secondary data analysis in systematic reviews.

The impact that a disability has on different aspects of life will vary widely from person to person, depending on factors such as the level of support from family, friends and carers. It is possible that there is no difference seen in children with learning disabilities as a group because these factors play more of a role in their oral health than their disability. In many ways this would be similar globally to CNLD as their socio-economic status seems to be one of the main factors in determining whether they experience dental caries and receive care.

The overall lower permanent tooth CI in CLD may be due to several barriers to treating those children; practitioners’ reluctance, lack of confidence, lack of training, lack of access to specialist provision, reluctance to refer children with significant additional care and support needs to GA, genuine difficulty, and a lack of behavioural and communication support. Although the data show a tendency for CLD to have their permanent teeth managed with more extractions than restoration treatments compared with CNLD, there is no further information from these studies on why this might be and again, it may be due to barriers to provision of care.

## Conclusions

- 1. Overall, there was no evidence that children with learning disabilities have different levels of dental caries in their permanent or primary dentition, to children without learning disabilities.
- 2. When the types of disabilities were separated out, there was evidence of lower levels of dental caries in children with Down syndrome in the permanent dentition, however, this could be linked to delayed tooth eruption. There was no evidence of a difference for children with autism or mixed learning disabilities.
- 3. There is some evidence of a difference in the amount and type of dental care provided for CLD based on quantitative (using the care and restorative indices) and narrative data, but this is sparse and this area should be strengthened by better reporting of datasets.

## Additional file

Additional file 1: Appendix 1: Newcastle-Ottawa Scale Risk of Bias Matrix; Appendix 2: data extraction tables from included studies. (DOCX 130 kb)

## Data Availability

The extracted data from individual papers within this review are available in Additional file 1: Appendix 2.
