# Supplementary material for: Dental caries experience, care index and restorative index in children with learning disabilities and children without learning disabilities; a systematic review and meta-analysis
Source: BMC Oral Health. 2019 Jul 15;19:146. doi: 10.1186/s12903-019-0795-4 (PMC6632188; doi:10.1186/s12903-019-0795-4)
Supplement: Supplementary file 1 — Appendix 1: Newcastle-Ottawa Scale Risk of Bias Matrix; Appendix 2: data extraction tables from included studies. (DOCX 130 kb) [file 12903_2019_795_MOESM1_ESM.docx]

| **Appendix 1A. Key Study Characteristics and Methodology: Down syndrome** | | | | | | | | | | | | |
| --- | --- | --- | --- | --- | --- | --- | --- | --- | --- | --- | --- | --- |
| **Study ID** | **Authors/ Title/ Citation** | **Year** | **Study Design** | **Study Characteristics** | **Number of clinical Examiners** | **Threshold for caries recording (e.g. D1, D2 etc.)** | **Caries detection/ recording system** | **Participant inclusion criteria** | **Participant exclusion criteria** | **Participant age range** | **Disability status** | **Sample size** |
| 1 | Al Habashneh, R  **Oral health status and reasons for not attending dental care among 12- to 16-year-old children with Down syndrome in special needs centres in Jordan**  Int J Dent Hygiene | 2012 | ***Study author’s designation:***  Non-specific “comparative study”  ***Review author’s designation:***  Comparison of two cross-sectional groups | ***Country:***  Jordan  ***Local/ National/ International:***  National  ***Clinical setting and related characteristics:***  Private dental clinic  ***Year study took place:***  *2008*  ***Control Group Definition:***  "matched non-DS/ public school children" aged 12-16 yrs | 1 | “frank carious cavitations on any surface of the tooth” | WHO | Down syndrome | Parental refusal | 12-16 years | Down syndrome | ***Total number of participants invited to take part:*** *study group – 150; control group – not specified*  ***Number of participants (study):*** *103*  ***Number of participants (control):*** *103*  ***Total number who took part:*** *206*  ***Reason for non-participation:*** *child unable to cooperate, parental refusal*  ***Was a sample size calculated?:*** *no* |
| 2 | Al Sarheed et al  **A comparative study of oral health amongst trisomy 21 children living in Riyadh, Saudi Arabia: Part 1 caries, malocclusion, trauma**  The Saudi Dental Journal | 2015 | ***Study author’s designation:***  Cross sectional study  ***Review author’s designation:***  Comparison of two cross-sectional groups | ***Country:***  Saudi Arabia  ***Local/ National/ International:***  Local  ***Clinical setting and related characteristics:***  Dental clinic at King Saud University  ***Year study took place:***  Not specified  ***Control Group Definition:***  Age and gender matched children without Down syndrome in schools throughout Riyadh | 2 | Not specified | WHO | Down syndrome | Not specified | 7-15 years | Down syndrome | ***Total number of participants invited to take part:*** not specified  ***Number of participants (study):*** 93  ***Number of participants (control):*** 99  ***Total number who took part:*** 192  ***Reason for non-participation:*** not specified  ***Was a sample size calculated?:*** no |
| 3 | Areias et al  **Does the chemistry in the saliva of Down syndrome children explain their low caries prevalence**  European Journal of Paediatric Dentistry | 2013 | ***Study author’s designation:***  “Sibling-matched population study”  ***Review author’s designation:***  Comparison of two cross-sectional groups | ***Country:***  Portugal  ***Local/ National/ International:***  National  ***Clinical setting and related characteristics:***  Not specified  ***Year study took place:***  *Not specified*  ***Control Group Definition:***  Children without Down syndrome with a DS sibling | Not specified | Not specified | DMFT | Down syndrome children included on a national database | systemic disease, previous medication within 3 weeks of saliva collection | 6-18 years | Down Syndrome | ***Total number of participants invited to take part:*** not specified  ***Number of participants (study):*** 45  ***Number of participants (control):*** 45  ***Total number who took part:*** 90  ***Reason for non-participation:*** not specified  ***Was a sample size calculated?:*** no |
| 4 | Cogulu, D et al  **Evaluation of the relationship between caries indices and salivary secretory IgA, salivary pH, buffering capacity and flow rate in children with Down Syndrome**  Archives of oral Biology | 2006 | ***Study author’s designation:***  Not specified  ***Review author’s designation:***  Comparison of two cross-sectional groups | ***Country:***  Turkey  ***Local/ National/ International:***  Local  ***Clinical setting and related characteristics:***  Dental clinic under natural light with a probe and mirror  ***Year study took place:***  Not specified  ***Control Group Definition:***  No DS, attending state school in Izmir, Turkey, living at home | 1 | Not specified | WHO | Down syndrome, non-institutionalised | Systemic disease, medication use in previous three months, no local infection in previous three months | 7-12 years | Down syndrome | ***Total number of participants invited to take part:*** not specified  ***Number of participants (study):*** 73  ***Number of participants (control):*** 70  ***Total number who took part:*** 143  ***Reason for non-participation:*** not specified  ***Was a sample size calculated?:*** no |
| 5 | Cornejo et al  **Bucodental health condition in patients with Down syndrome of Cordoba City, Argentina**  Acta odontológica latinoamericana | 1996 | ***Study author’s designation:***  Non-specific study with “control group”  ***Review author’s designation:***  Comparison of two cross-sectional groups | ***Country:***  Argentina  ***Local/ National/ International:***  Local  ***Clinical setting and related characteristics:***  School classroom in natural light with mouth mirror and ‘explorer’  ***Year study took place:***  *1993*  ***Control Group Definition:***  Healthy children of the same age in primary and secondary schools in Cordoba | 1 | Not specified | WHO | Down syndrome | Not specified | 3-19 years | Down syndrome | ***Total number of participants invited to take part:*** not specified  ***Number of participants (study):*** 86  ***Number of participants (control):*** 86  ***Total number who took part:*** 172  ***Reason for non-participation:*** not specified  ***Was a sample size calculated?:*** no |
| 6 | Davidovich et al  **A comparison of the sialochemistry, oral pH, and oral health status of down syndrome children to healthy children**  International Journal of Paediatric Dentistry | 2010 | ***Study author’s designation:***  Not specified  ***Review author’s designation:***  Comparison of two cross-sectional groups | ***Country:***  Israel  ***Local/ National/ International:***  Local  ***Clinical setting and related characteristics:***  Dental clinic under natural light with mirror and probe  ***Year study took place:***  Not specified  ***Control Group Definition:***  No DS, attending the Hebrew University Hadassah School of Dental Medicine, Israel | 2 | Not specified | WHO | Down syndrome | Not specified | 1-9 years | Down syndrome | ***Total number of participants invited to take part:*** not specified  ***Number of participants (study):*** 70  ***Number of participants (control):*** 32  ***Total number who took part:*** 102  ***Reason for non-participation:*** not specified  ***Was a sample size calculated?:*** no |
| 7 | Hashizume et al.,  **Salivary secretory IgA concentration and dental caries in children with Down syndrome**  Special Care Dentistry | 2017 | ***Study author’s designation:***  Non-specific study “with a control group”  ***Review author’s designation:***  Comparison of two cross-sectional groups | ***Country:***  Brazil  ***Local/ National/ International:***  Local  ***Clinical setting and related characteristics:***  Chair under artificial light by a calibrated examiner using a mouth mirror and blunt dental probe  ***Year study took place:***  *Not specified*  ***Control Group Definition:***  Children without Down syndrome attending a public primary school in Porto Algere | 1 | carious lesions at the cavitation stage | DMFT | Down syndrome | Use of orthodontic appliances, presence of systematic disease, inability to provide a saliva sample, failure to cooperate with examination. | 6-14 years | Down syndrome | ***Total number of participants invited to take part:*** not specified  ***Number of participants (study):*** 61  ***Number of participants (control):*** 52  ***Total number who took part:*** 113  ***Reason for non-participation:*** not specified  ***Was a sample size calculated?:*** no |
| 8 | Lee et al  **Dental caries and salivary immunoglobulin A in Down syndrome children**  Journal of paediatric Child health | 2004 | ***Study author’s designation:***  Not specified  ***Review author’s designation:***  Comparison of two cross-sectional groups | ***Country:***  Korea  ***Local/ National/ International:***  Not specified  ***Clinical setting and related characteristics:***  Not specified  ***Year study took place:***  Not specified  ***Control Group Definition:***  Children without DS, attending schools in same regions as study population, matched for age, gender and region | 1 | Not specified | National Institute for Dental Research (NIDR) | Down syndrome | No previous significant disease, no medication use in previous two weeks | 8-17 years | Down syndrome | ***Total number of participants invited to take part:*** not specified  ***Number of participants (study):*** 19  ***Number of participants (control):*** 41  ***Total number who took part:*** 60  ***Reason for non-participation:*** "inappropriate saliva sample collection"  ***Was a sample size calculated?:*** no |
| 9 | Mathias, M et al  **Some factors associated with dental caries**  **in the primary dentition of children**  **with Down syndrome**  European Journal of Paediatric Dentistry | 2011 | ***Study author’s designation:***  Not specified  ***Review author’s designation:***  Comparison of two cross-sectional groups | ***Country:***  Brazil  ***Local/ National/ International:***  Local  ***Clinical setting and related characteristics:***  Dental office under artificial light  ***Year study took place:***  Not specified  ***Control Group Definition:*** No Down syndrome at kindergarten in Baurau, brazil | 1 | Not specified | DMFT | Down syndrome | Not specified | 1-7 years | Down syndrome | ***Total number of participants invited to take part:*** not specified  ***Number of participants (study):*** 69  ***Number of participants (control):*** 69  ***Total number who took part:*** 138  ***Reason for non-participation:*** not specified  ***Was a sample size calculated?:*** no |
| 10 | Stabholz et al    **Caries experience, periodontal treatment needs, salivary PH, and Streptococcus mutans counts in preadolescent Down syndrome population**  Special Care in Dentistry | 1991 | ***Study author’s designation:***  Non-specific study “with two control groups”  ***Review author’s designation:***  Comparison of two cross-sectional groups | ***Country:***  Israel  ***Local/ National/ International:***  Local  ***Clinical setting and related characteristics:***  Not specified  ***Year study took place:***  Not specified  ***Control Group Definition:***  Healthy children from a local elementary school | 1 | Not specified | WHO | Down syndrome children living in the Elwin Institute, Jerusalem | Not specified | 8-13 years | Down syndrome | ***Total number of participants invited to take part:*** not specified  ***Number of participants (study):*** 32  ***Number of participants (control):*** 30  ***Total number who took part:*** 62  ***Reason for non-participation:*** not specified  ***Was a sample size calculated?:*** no |
| 11 | Subramanium, P et al  **Assessment of salivary total antioxidant levels and oral health status in children with Down syndrome**  Special Care Dentistry | 2014 | ***Study author’s designation:***  Not specified  ***Review author’s designation:***  Comparison of two cross-sectional groups | ***Country:***  India  ***Local/ National/ International:***  Local  ***Clinical setting and related characteristics:***  ***School***  ***Year study took place:***  *Not specified*  ***Control Group Definition:***  Healthy children visiting the department of paedodontics and preventive dentistry | 1 | Not specified | WHO | Down syndrome, institutionalised | Associated medical conditions, long term medication, inability to tolerate examination, lack of written parental consent | 7-12 years | Down syndrome | ***Total number of participants invited to take part:*** not specified  ***Number of participants (study):*** 34  ***Number of participants (control):*** 34  ***Total number who took part:*** 68  ***Reason for non-participation:*** not specified  ***Was a sample size calculated?:*** no |
| **Appendix 1B. Key Study Characteristics and Methodology: Autism** | | | | | | | | | | | | |
|  | **Authors/ Title/ Citation** | **Year** | **Study Design** | **Study Characteristics** | **Number of Clinical Examiners** | **Threshold for caries recording (e.g. D1, D2 etc.)** | **Caries detection/ recording system** | **Participant inclusion criteria** | **Participant exclusion criteria** | **Participant age range** | **Disability status** | **Sample size** |
| 12 | Al-Maweri et al  **Oral lesions and dental status of autistic childfren in Yemen: A case-control study**  Journal of International Society of Preventive & Community Dentistry | 2014 | ***Study author’s designation:***  Case-control study  ***Review author’s designation:***  Comparison of two cross-sectional groups | ***Country:***  Yemen  ***Local/ National/ International:***  Local  ***Clinical setting and related characteristics:***  School, overhead light with tweezers, gauze, mirror and tongue depressor  ***Year study took place:***  2013  ***Control Group Definition:***  children without autism, age and gender matched from two public schools in same neighborhood | 1 | Not specified | WHO | Children with autism attending the Al-Yemen special centre for rehabilitation and education of children with autism in Sana'a. Children had to be cooperative for examination and have a basic understanding of very simple instructions. | Other systemic disease known to cause dental problems and children unable to cooperate for examination. | 5-16 years | Autism | ***Total number of participants invited to take part:*** Not specified  ***Number of participants (study):*** 42  ***Number of participants (control):*** 84  ***Total number who took part:*** 126  ***Reason for non-participation:*** Did not meet inclusion criteria  ***Was a sample size calculated?:*** No |
| 13 | Bhandary et al  **Salivary biomarker levels and oral health status of children with autistic spectrum disorders: a comparative study**  European Archives of Paediatric Dentistry | 2017 | ***Study author’s designation:***  Sibling-matched control study  ***Review author’s designation:***  Comparison of two cross-sectional groups | ***Country:***  India  ***Local/ National/ International:***  Local  ***Clinical setting and related characteristics:***  Not specified  ***Year study took place:***  Not specified  ***Control Group Definition:***  Healthy siblings of the ASD children | 1 | Not specified | WHO | ASD diagnosis by neurologist | uncooperative children, healthy siblings with underlying medical condition, lack of parental consent | 6-12 years | Autism | ***Total number of participants invited to take part:*** Not specified  ***Number of participants (study):*** 30  ***Number of participants (control):*** 30  ***Total number who took part:*** 60  ***Reason for non-participation:*** Not specified  ***Was a sample size calculated?:*** Yes (no further information) |
| 14 | Du et al  **"Oral health among preschool children with autism spectrum disorders:**  **A case-control study"**  Autism | 2014 | ***Study author’s designation:***  Case control study  ***Review author’s designation:***  Comparison of two cross-sectional groups | ***Country:***  Hong Kong  ***Local/ National/ International:***  National  ***Clinical setting and related characteristics:***  School, examinations in a chair with a mirror with built-in LED  ***Year study took place:***  Not specified  ***Control Group Definition:***  No ASD, attending mainstream schools in Hong Kong | 1 | Not specified | WHO | ASD, attending special child care centres in Hong Kong | Lack of parental consent, unfeasibility of oral examination | 3-7 years | Autism | ***Total number of participants invited to take part:*** Not specified  ***Number of participants (study):*** 257  ***Number of participants (control):*** 257  ***Total number who took part:*** Not specified  ***Reason for non-participation:*** Lack of parental consent and children unable to tolerate examination  ***Was a sample size calculated?:*** Yes, 243, 80% power |
| 15 | El Khatib et al  **Oral health status and behaviours of children with Autism Spectrum Disorder: a case-control study**  International Journal of Paediatric Dentistry | 2014 | ***Study author’s designation:***  Case control study  ***Review author’s designation:***  Comparison of two cross-sectional groups | ***Country:***  Egypt  ***Local/ National/ International:***  Local  ***Clinical setting and related characteristics:***  Institutions/schools, examinations on chairs, tables or knee-to-knee  ***Year study took place:***  2009-2011  ***Control Group Definition:***  healthy children from government or private schools, matched for age, sex, socioeconomic status | 1 | Not specified | WHO | Autistic spectrum disorder (ASD) in government or private institutions of intellectually disabled children in Alexandria | No parental consent | 3-13 years | Autism | ***Total number of participants invited to take part:*** not specified  ***Number of participants (study):*** 100  ***Number of participants (control):*** 100  ***Total number who took part:*** 200  ***Reason for non-participation:*** Not specified  ***Was a sample size calculated?:*** Yes - assumptions made for sample size estimation were as follows; caries prevalence among healthy children = 40%, caries prevalence among ASD children = 60%, alpha error = 5%, beta error=20%. MedCalc software estimated sample size per group = 94 (~100 children per group) |
| 16 | Fakroon et al  **Dental caries experience and periodontal treatment needs of children with autistic spectrum disorder**  European Archives of Paediatric Dentistry | 2014 | ***Study author’s designation:***  Cross sectional, comparative case-control study  ***Review author’s designation:***  Comparison of two cross-sectional groups | ***Country:***  Libya  ***Local/ National/ International:***  Local  ***Clinical setting and related characteristics:***  Classroom under natural and artificial light  ***Year study took place:***  2013  ***Control Group Definition:***  Children without ASD, attending schools in same area as the study population | 2 | A tooth with definite cavity, undermined enamel, or detectably softened or leathery area of enamel or cementum. In cases where the tooth’s crown was entirely destroyed by caries leaving only the root, the caries was judged to have originated on the crown.  • A tooth with a temporary filling.  • A tooth that was sealed but decayed.  • Filled tooth with additional decay. | WHO | ASD, attending Benghazi Centre of Autism | Inability to tolerate examination, lack of written parental consent | 3-14 years | Autism | ***Total number of participants invited to take part:*** 105  ***Number of participants (study):*** 50  ***Number of participants (control):*** 50  ***Total number who took part:*** 100  ***Reason for non-participation:*** Lack of parental consent and children unable to tolerate examination  ***Was a sample size calculated?:*** Yes, 50 children in each group for 80% power and 95% CI |
| 17 | Jaber et al  **Dental caries experience, oral health status and treatment needs of dental patients with autism**  Journal of Applied Oral Science | 2011 | ***Study author’s designation:***  Non-specific study “with a control group”  ***Review author’s designation:***  Comparison of two cross-sectional groups | ***Country:***  UAE  ***Local/ National/ International:***  Local  ***Clinical setting and related characteristics:***  Dental chair under flash light/natural light with mirror and probes  ***Year study took place:***  Not specified  ***Control Group Definition:***  non-autistic children, mainly relatives of study subjects, matched for age, sex and socio-economic status and dental care background | 1 | Not specified | WHO | Autistic, attending Dubai and Sharjah Autism Centres | Dental prophylaxis in last six months, suffering from other conditions known to affect caries (e.g. DS) | 6-16 years | Autism | ***Total number of participants invited to take part:*** Not specified  ***Number of participants (study):*** 61  ***Number of participants (control):*** 61  ***Total number who took part:*** 122  ***Reason for non-participation:*** Not specified  ***Was a sample size calculated?:*** No |
| 18 | Namal et al  **Do autistic children have higher levels of caries? A cross sectional study in Turkish children**  Journal of Indian Society of Pedodontics and Preventive Dentistry | 2007 | ***Study author’s designation:***  Cross sectional study  ***Review author’s designation:***  Comparison of two cross-sectional groups | ***Country:***  Turkey  ***Local/ National/ International:***  Local  ***Clinical setting and related characteristics:***  School classroom with mirror and natural daylight or portable light source  ***Year study took place:***  Not specified  ***Control Group Definition:***  Healthy children in three randomly selected schools in neighboring regions | 1 | Not specified | WHO | ASD children from 3 schools; 1 autistic centre and 2 elementary schools | Not specified | 7-12 years | Autism | ***Total number of participants invited to take part:*** Not specified  ***Number of participants (study):*** 62  ***Number of participants (control):*** 301  ***Total number who took part:*** 363  ***Reason for non-participation:*** Not specified  ***Was a sample size calculated?:*** No |
| 19 | Yashoda et al  **Oral health status and parental perception of child oral health related quality-of-life of children with autism in Bangalore, India**  Journal of Indian Society of Pedodontics and Preventive Dentistry | 2014 | ***Study author’s designation:***  Comparative cross-sectional study  ***Review author’s designation:***  Comparison of two cross-sectional groups | ***Country:***  India  ***Local/ National/ International:***  Local  ***Clinical setting and related characteristics:***  School, seated on a chair under natural light with dental instruments  ***Year study took place:***  2012  ***Control Group Definition:***  Children without Autism from regular schools | 1 | Not specified | DMFT | Diagnosis of autism based on records. | Oral prophylaxis in the last 6 months, other diseases known to influence caries or periodontal disease. Those undergoing antibiotic or anti-inflammatory therapy in the last 6 months. | 4-15 years | Autism | ***Total number of participants invited to take part:*** Not specified  ***Number of participants (study):***135  ***Number of participants (control):*** 135  ***Total number who took part:*** 270  ***Reason for non-participation:*** Not specified  ***Was a sample size calculated?:*** No |
| **Appendix 1C. Key Study Characteristics and Methodology: Mixed or Non-Specific Learning Disabilities** | | | | | | | | | | | | |
|  | **Authors/ Title/ Citation** | **Year** | **Study Design** | **Study Characteristics** | **Number of Clinical Examiners** | **Threshold for caries recording (e.g. D1, D2 etc.)** | **Caries detection/ recording system** | **Participant inclusion criteria** | **Participant exclusion criteria** | **Participant age range** | **Disability status** | **Sample size** |
| 20 | Bakry et al  **Risk factors associated with caries experience in children and adolescents with intellectual disabilities**  The Journal of Clinical Pediatric Dentistry | 2012 | ***Study author’s designation:***  Not specified  ***Review author’s designation:***  Comparison of two cross-sectional groups | ***Country:***  Saudi Arabia  ***Local/ National/ International:***  Local  ***Clinical setting and related characteristics:***  Clinical setting not specified. Use of mouth mirror, dental probe and “optimal lighting”  ***Year study took place:***  Not specified  ***Control Group Definition:***  Children without intellectual disability | 2 | Frank carious lesion detection | WHO | Diagnosis with any level of intellectual disability | Lack of parental consent | 3-13 years | Intellectual disability | ***Total number of participants invited to take part:*** Not specified  ***Number of participants (study):*** 33  ***Number of participants (control):*** 53  ***Total number who took part:*** 86  ***Reason for non-participation:***  Not specified  ***Was a sample size calculated?:*** No |
| 21 | Forsberg et al  **Dental health and dental care in severely mentally retarded children**  Swedish Dental Journal | 1985 | ***Study author’s designation:***  Non-specific study with a matched control group”  ***Review author’s designation:***  Comparison of two cross-sectional groups | ***Country:***  Sweden  ***Local/ National/ International:***  Local  ***Clinical setting and related characteristics:***  Paediatric clinic for children with severe mental retardation (SMR) in Umea, Sweden. No info. On control group  ***Year study took place:***  1977  ***Control Group Definition:***  A healthy child of normal intelligence, of the same sex and place of residence, and 22with a date of birth as near to that of the mentally retarded child as possible" | 1 | Clinically, anything beyond a WSL was recorded, radiographically lesions > half way through enamel were recorded. | DMFT | SMR children born between '59-'73 living in medical services district of Umea, Sweden. | Not specified | 3-17 years | Severe mental retardation | ***Total number of participants invited to take part:*** 110 SMR, 108 control children  ***Number of participants (study):*** 100  ***Number of participants (control):*** 103  ***Total number who took part:*** 203  ***Reason for non-participation:***  7 registered SMR children had left the district, one was deceased, 2 were unable to cooperate with examination, 5 controls unwilling to participate  ***Was a sample size calculated?:*** No |
| 22 | Jokic et al  **Dental caries in disabled children**  Collegium Antropologicum | 2007 | ***Study author’s designation:***  Not specified  ***Review author’s designation:***  Comparison of two cross-sectional groups | ***Country:***  Croatia  ***Local/ National/ International:***  Local  ***Clinical setting and related characteristics:***  Not specified  ***Year study took place:***  Not specified  ***Control Group Definition:***  Healthy children | Not specified | Clinically detected cavitations | DMFT | Disabled children from centres for rehabilitation | Not specified | 3-17 years | Cerebral Palsy, Down syndrome, mental retardation, autism, hearing/speaking disorders | ***Total number of participants invited to take part:*** Not specified  ***Number of participants (study):*** 80  ***Number of participants (control):*** 80  ***Total number who took part:*** 160  ***Reason for non-participation:***  Not specified  ***Was a sample size calculated?:*** No |
| 23 | Moreira et al  **Does intellectual disability affect the development of dental caries in patients with cerebral palsy?**  Research in Developmental Disabilities | 2012 | ***Study author’s designation:***  Cross sectional study  ***Review author’s designation:***  Comparison of two cross-sectional groups | ***Country:***  Brazil  ***Local/ National/ International:***  Local  ***Clinical setting and related characteristics:***  Clinical setting using clinical probes and mouth mirrors  ***Year study took place:***  Not specified  ***Control Group Definition:***  Children without CP matched for age, gender and sex attending a regular public school | 3 | Not specified | WHO | Diagnosis of spastic cerebral palsy attending a physical rehabilitation centre. | Not specified | Not specified. Average age of participants was 8.9 years | Cerebral palsy with intellectual disability | ***Total number of participants invited to take part:*** Not specified  ***Number of participants (study):*** 76  ***Number of participants (control):*** 89  ***Total number who took part:*** 165  ***Reason for non-participation:***  Not specified  ***Was a sample size calculated?:*** Yes - 59 participants per group, adjusted for loss to follow up. |
| 24 | Palin et al  **Dental health of 9-10-year-old mentally retarded children in Eastern Finland**  Community Dentistry and oral Epidemiology | 1981 | ***Study author’s designation:***  Not specified  ***Review author’s designation:***  Comparison of two cross-sectional groups | ***Country:***  Finland  ***Local/ National/ International:***  Regional  ***Clinical setting and related characteristics:***  Not specified  ***Year study took place:***  1978  ***Control Group Definition:***  Children in Finland with “normal mental performance” | 1 | Grade 3 & 4 by Moller and Poulsen criteria | Moller and Poulsen | Mentally retarded | Not specified | 9-10 years | Mental retardation | ***Total number of participants invited to take part:*** Not specified  ***Number of participants (study):*** 58  ***Number of participants (control):*** 58  ***Total number who took part:*** 116  ***Reason for non-participation:***  Failure to attend, inability to contact family, illness, moving away from the area before examination  ***Was a sample size calculated?:*** No |
| 25 | Pope et al  **The dental status of cerebral palsied children**  Pediatric dentistry | 1991 | ***Study author’s designation:***  Matched control group study  ***Review author’s designation:***  Comparison of two cross-sectional groups | ***Country:***  UK  ***Local/ National/ International:***  Local  ***Clinical setting and related characteristics:***  Not specified  ***Year study took place:***  Not specified  ***Control Group Definition:***  Healthy children matched to the study group for race, age and social class | Not specified | Not specified | Rradike (1972) | Cerebral Palsy, with or without mental handicap, attending special schools in Leeds, England | Not specified | 3-18 years | Cerebral Palsy | ***Total number of participants invited to take part:*** Not specified  ***Number of participants (study):*** 50 (11 years old and over, other age groups within study population not broken down to LD and non-LD)  ***Number of participants (control):*** 95  ***Total number who took part:*** 145  ***Reason for non-participation:***  Not specified  ***Was a sample size calculated?:*** No |

| **Appendix 2A. Study Results: Down syndrome** | | | | | | | | | | | | | | |
| --- | --- | --- | --- | --- | --- | --- | --- | --- | --- | --- | --- | --- | --- | --- |
| **Study ID** | **Authors/ Title/ Citation** | **Year** | **CI (with disability)** | **RI (with disability)** | **DMFT (with disabilities)** | **D (with disability)** | **M (with disability)** | **F (with disability)** | **CI (control)** | **RI (control)** | **DMFT (control)** | **D (control)** | **M (control)** | **F (control)** |
| 1 | Al Habashneh, R  **Oral health status and reasons for not attending dental care among 12- to 16-year-old children with Down syndrome in special needs centres in Jordan**  Int J Dent Hygiene | 2012 | not available | not available | DMFT 3.32  (SD 3.77) | not available | not available | not available | not available | not available | DMFT 4.59  (SD 4.21) | not available | not available | not available |
| 2 | Al Sarheed et al  **A comparative study of oral health amongst trisomy 21 children living in Riyadh, Saudi Arabia: Part 1 caries, malocclusion, trauma**  The Saudi Dental Journal | 2015 | 0.18 | 0.22 | DMFT 2.66  (SD 3.09) | 2.11 | 0.29 | 0.52 | 0.18 | 0.19 | DMFT 3.11  (SD 2.58) | 2.37 | 0.19 | 0.55 |
| 3 | Areias et al  **Salivary factors on Down syndrome children**  European Journal of Paediatric Dentistry | 2013 | 0.16 | 0.27 | DMFT 1.02  (SD 2.42) | 0.44 | 0.42 | 0.16 | 0.02 | 0.04 | DMFT 1.84  (SD 3.13) | 0.87 | 0.93 | 0.04 |
| 4 | Cogulu, D et al  **Evaluation of the relationship between caries indices and salivary secretory IgA, salivary pH, buffering capacity and flow rate in children with Down Syndrome**  Archives of oral Biology | 2006 | not available | not available | DMFS 0.92 | not available | not available | not available | not available | not available | DMFS 4.26 | not available | not available | not available |
| 5 | Cornejo et al  **Bucodental health condition in patients with Down syndrome of Cordoba City, Argentina**  Acta odontológica latinoamericana | 1996 | not available | not available | dmft  3-6yrs =2.8  (SD 0.8)  **7-9yrs = 2.4***  **(SD 0.6)**  10-13yrs = 1.1  (SD0.4)  14-16yrs = 0.5  (SD 0.2)  17-19 = 0.8  (SD 0.2)  DMFT  3-6yrs=0.1  (SD 0.04)  7-9yrs= 0.9  (SD 0.2)  **10-13yrs=1.3**  **(SD 0.3)***  14-16yrs= 1.3  (SD 0.4)  17-19=4.6  (SD 0.7) | not available | not available | not available | not available | not available | dmft  3-6= 0.8  (SD 0.4)  **7-9 = 1.7**  **(SD 0.3)***  DMFT  3-6yrs = 0.4  (SD 0.01)  7-9yrs = 0.6  (SD 0.2)  **10-13yrs= 1.7***  **(SD 0.4)**  14-16yrs = 2.0 (SD 0.5)  17-19yrs = 4.9  (SD 0.8) | not available | not available | not available |
| 6 | Davidovich et al  **A comparison of the sialochemistry, oral pH, and oral health status of down syndrome children to healthy children**  International Journal of Paediatric Dentistry | 2010 | not available | not available | DMFT 3.37  (SD 0.56) | not available | not available | not available | not available | not available | DMFT 5.9  (SD 0.80) | not available | not available | not available |
| 7 | Hashizume et al.  **Salivary secretory IgA concentration and dental caries in children with Down syndrome**  Special Care Dentistry | 2017 | not available | not available | dmft 1.84  (SD 3.67)  DMFT 0.36  (SD 1.00) | not available | not available | not available | not available | not available | dmft 0.98  (SD 1.39)  DMFT 0.4  (SD 0.92) | not available | not available | not available |
| 8 | Lee et al  **Dental caries and salivary immunoglobulin A in Down syndrome children**  Journal of paediatric Child health | 2004 | not available | not available | DMFS 4.82  (SD 5.64) | not available | not available | not available | not available | not available | DMFS 8.35  (SD 6.25) | not available | not available | not available |
| 9 | Mathias, M et al  **Some factors associated with dental caries**  **in the primary dentition of children**  **with Down syndrome**  European Journal of Paediatric Dentistry | 2011 | not available | not available | DMFT 2.2  (SD 6.3) | not available | not available | not available | not available | not available | DMFT 3.4  (SD 8.1) | not available | not available | not available |
| 10 | Stabholz et al    **Caries experience, periodontal treatment needs, salivary PH, and Streptococcus mutans counts in preadolescent Down syndrome population**  Special Care in Dentistry | 1991 | not available | not available | DMFS 1.2** | not available | not available | not available | not available | not available | DMFS 14.5** | not available | not available | not available |
| 11 | Subramanium, P et al  **Assessment of salivary total antioxidant levels and oral health status in children with Down syndrome**  Special Care Dentistry | 2014 | not available | not available | DMFT=1.68  (SD 0.69)  deft=2.69  (SD 1.62) | not available | not available | not available | not available | not available | DMFT=1.84  (SD 1.12)  deft=2.90  (SD 1.60) | not available | not available | not available |
|  | **Appendix 2B. Study Results: Autism** | | | | | | | | | | | | | |
| **Study ID** | **Authors/ Title/ Citation** | **Year** | **CI (with disability)** | **RI (with disability)** | **DMFT (with disabilities)** | **D (with disability)** | **M (with disability)** | **F (with disability)** | **CI (control)** | **RI (control)** | **DMFT (control)** | **D (control)** | **M (control)** | **F (control)** |
| 12 | Al-Maweri et al  **Oral lesions and dental status of autistic childfren in Yemen: A case-control study**  Journal of International Society of Preventive & Community Dentistry | 2014 | permanent=0.03, primary=0.05 | 0.04 | dmft=5.23  (SD 2.34)  DMFT=2.0  (SD 2.18) | 1.86 | 0.05 | 0.05 | Permanent =0.02, primary=0.1 | 0.03 | dmft=4.06  (SD 2.98)  DMFT=1.27  (SD 1.77) | 1.22 | 0.03 | 0.03 |
| 13 | Bhandary et al  **Salivary biomarker levels and oral health status of children with autistic spectrum disorders: a comparative study**  European Archives of Paediatric Dentistry | 2017 | 0 | 0 | DMFT=0.37  (SD 0.62) | 0.27 | 0.1 | 0 | 0.19 | 0.35 | DMFT=0.37  (SD 0.56) | 0.13 | 0.17 | 0.07 |
| 14 | Du et al  **"Oral health among preschool children with autism spectrum disorders:**  **A case-control study"**  Autism | 2014 | 0.1 | 0.11 | dmfs 3.73  (SD 9.03) | 3.14 | 0.21 | 0.38 | 0.09 | 0.09 | dmfs 5.41  (SD 9.18) | 4.31 | 0.09 | 0.41 |
| 15 | El Khatib et al  **Oral health status and behaviours of children with Autism Spectrum Disorder: a case-control study**  International Journal of Paediatric Dentistry | 2014 | not available | not available | **primary dentition dmft=3.53**  **(SD 4.57)***  Mixed  DFT=3.33  (SD 3.39)  DMFT = 0.93 (SD 1.58)  **permanent DMFT 3.4**  **(SD 4.54)*** | 2.98 mixed dentition | not available | ASD=0.37 (primary), mixed = 0.35 | not available | not available | **primary dmft**  **3.56**  **(SD 3.86)***  mixed  DFT 2.94  (SD 2.94)  DMFT 0.79 (SD 1.26)  **Permanent DMFT 3.50 (SD 3.63)*** | mixed = 1.85 | not available | 0.85 (primary dentition) , mixed = 1.1 |
| 16 | Fakroon et al  **Dental caries experience and periodontal treatment needs of children with autistic spectrum disorder**  European Archives of Paediatric Dentistry | 2014 | permanent teeth: CI=0.10, deciduous teeth: CI=0.25 | permanent teeth: RI=0.10, deciduous teeth: RI=0.25 | dmft 1.13  (SD 1.84)  DMFT 0.22  (SD 0.08) | D:0.20, d:0.85 | M:0.00, m:0.02 | F:0.02, f:0.29 | permanent teeth: CI=0.06, deciduous teeth: CI=0.02 | permanent teeth: RI-=0.07, deciduous teeth: RI=0.03 | dmft:2.85  (SD 3.32)  DMFT  1.15  (SD 0.27) | D:1.00, d:2.65 | M:0.07, m:0.13 | F:0.07, f:0.07 |
| 17 | Jaber et al  **Dental caries experience, oral health status and treatment needs of dental patients with autism**  Journal of Applied Oral Science | 2011 | not available | 0.02 | dmft 0.8  (SD 0.2)  DMFT 1.6  (SD 0.64) | not available | not available | not available | not available | not available | dmft 0.3  (SD 0.3)  DMFT 0.6  (SD 0.29) | not available | not available | not available |
| 18 | Namal et al  **Do autistic children have higher levels of caries? A cross sectional study in Turkish children**  Journal of Indian Society of Pedodontics and Preventive Dentistry | 2007 | 0.04 | 0.06 | DMFT = 1.74** | 1.08 | 0.56 | 0.06 | 0.05 | 0.06 | DMFT = 2.41** | 2.27 | 0.02 | 0.14 |
| 19 | Yoshoda et al  **Oral health status and parental perception of child oral health related quality-of-life of children with autism in Bangalore, India**  Journal of Indian Society of Pedodontics and Preventive Dentistry | 2014 | not available | not available | dmft 0.4  (SD 2.48)  DMFT 0.86 (SD 1.22) | not available | not available | not available | not available | not available | dmft=0.59 (SD1.28)  DMFT 0.46 (SD1.06) | not available | not available | not available |
|  | **Appendix 2C. Study Results: Mixed or Non-Specific Learning Disabilities** | | | | | | | | | | | | | |
| **Study ID** | **Authors/ Title/ Citation** | **Year** | **CI (with disability)** | **RI (with disability)** | **DMFT (with disabilities)** | **D (with disability)** | **M (with disability)** | **F (with disability)** | **CI (control)** | **RI (control)** | **DMFT (control)** | **D (control)** | **M (control)** | **F (control)** |
| 20 | Bakry et al  **Risk factors associated with caries experience in children and adolescents with intellectual disabilities**  The Journal of Clinical Pediatric Dentistry | 2012 | not available | permanent=0.12, primary=0.05 | dft 6.81 (SD6.11)  DFT=2.32 (SD2.98) | 2.04 | not available | 0.28 | not available | permanent=0.84, primary=0.04 | dft 8.83 (SD4.99)  DFT 0.92 (SD 1.57) | 0.77 | not available | 0.15 |
| 21 | Forsberg et al  **Dental health and dental care in severely mentally retarded children**  Swedish Dental Journal | 1985 | not available | not available | deft given  3-5yrs 1.3  (SD 2.2)  6-11yrs 2.4  (SD 3.1)  **3-11yrs 2.0**  **(SD 2.9)***  DMFT  6-11yrs 2.1  (SD 4.0)  **12-17yrs 7.2**  **(SD 6.1)***  6-17yrs 5.3  (SD 5.9) | not available | not available | not available | not available | not available | deft  3-5yrs 2.5  (SD 2.6)  6-11yrs 3.7  (SD 2.8)  **3-11yrs 3.3**  **(SD 2.8)***  DMFT  6-11yrs 2.4  (SD 2.0)  **12-17yrs 9.0**  **(SD 4.0)***  6-17yrs 6.5  (SD 4.7) | not available | not available | not available |
| 22 | Jokic et al  **Dental caries in disabled children**  Collegium Antropologicum | 2007 | not available | not available | dft 3.42**  DMFT 6.39** | not available | not available | not available | not available | not available | dft 1.43**  DMFT 4.76** | not available | not available | not available |
| 23 | Moreira et al  **Does intellectual disability affect the development of dental caries in patients with cerebral palsy?**  Research in Developmental Disabilities | 2012 | not available | not available | DMFT 5.2  (SD 5.75) | not available | not available | not available | not available | not available | DMFT 1.5  (SD 2.1) | not available | not available | not available |
| 24 | Palin et al  **Dental health of 9-10-year-old mentally retarded children in Eastern Finland**  Community Dentistry and oral Epidemiology | 1981 | not available | not available | not available | not available | not available | not available | not available | not available | not available | not available | not available | not available |
| 25 | Pope et al  **The dental status of cerebral palsied children**  Pediatric dentistry | 1991 | 0.36 | 0.43 | DMFT 2.94** | 1.4 | 0.48 | 1.06 | 0.82 | 0.82 | DMFT 2.27** | 0.4 | 0 | 1.8 |

* Indicates the age group chosen for analysis where there was a choice and no overall dmft or DMFT was given

**No standard deviation given in paper or calculatable

| **Appendix 3A. Findings and Quality Appraisal: Down syndrome** | | | | | | | | |
| --- | --- | --- | --- | --- | --- | --- | --- | --- |
| **Study ID** | **Authors/ Title/ Citation** | **Year** | **Study Author Conclusions** | **Risk of Bias (RoB) Assessment (Newcastle-Ottawa Scale)** | | | | **Review Author Additional Notes** |
|  |  |  |  | **Selection**  **(maximum 5 points)** | **Comparability**  **(maximum 2 points)** | **Outcome**  **(maximum 3 points)** | **RoB** |  |
| 1 | Al Habashneh, R  **Oral health status and reasons for not attending dental care among 12- to 16-year-old children with Down syndrome in special needs centres in Jordan**  Int J Dent Hygiene | 2012 | "less dental attendance" in DS children and "the mean DMFT … was higher in non-DS children than in DS children" | 2 | 2 | 2 |  |  |
| 2 | Al Sarheed et al  **A comparative study of oral health amongst trisomy 21 children living in Riyadh, Saudi Arabia: Part 1 caries, malocclusion, trauma**  The Saudi Dental Journal | 2015 | No stat sig difference in caries incidence between groups, DS children more likely to have class III malocclusions and incisor trauma. Early interventive prevention may preclude the incidence of incisal fractures. | 3 | 1 | 2 |  |  |
| 3 | Areias et al  **Salivary factors on Down syndrome children**  European Journal of Paediatric Dentistry | 2013 | No correlation between salivary composition and dental caries in DS children | 1 | 1 | 1 |  |  |
| 4 | Cogulu, D et al  **Evaluation of the relationship between caries indices and salivary secretory IgA, salivary pH, buffering capacity and flow rate in children with Down Syndrome**  Archives of oral Biology | 2006 | DS have a reduced caries experience compared with non-DS children. Stat sig negative correlation between dental caries and salivary IgA (higher IgA levels may be caries-protective and may be a further reason (agenesis, microdontia, delayed eruption) as to why DS children have a reduced caries experience. | 3 | 1 | 2 |  |  |
| 5 | Cornejo et al  **Bucodental health condition in patients with Down syndrome of Cordoba City, Argentina**  Acta odontológica latinoamericana | 1996 | Caries experience is very similar between DS and non-DS groups however DS children receive less treatment of the deciduous dentition - this may be due to the delay in eruption of the teeth when examined alongside non-DS children of a similar age. | 1 | 2 | 1 |  |  |
| 6 | Davidovich et al  **A comparison of the sialochemistry, oral pH, and oral health status of down syndrome children to healthy children**  International Journal of Paediatric Dentistry | 2010 | Both pH and sialochemistry (Cl, Ca, Na and K levels) were different among children with DS with caries compared to healthy children.  The differences between DS and healthy children were more prominent in relation to caries status.  All variables, taken together, could not exactly explain the overall low caries rate of children with DS, thus only demonstrates the complex nature of dental caries. We believe that the trisomy in DS manifests itself in the salivary glands. As a result, a different salivary environment of electrolytes is created, that interferes in the caries process, leading to lower caries rates among DS children. | 0 | 1 | 2 |  |  |
| 7 | Hashizume et al.,  **Salivary secretory IgA concentration and dental caries in children with Down syndrome**  Special Care Dentistry | 2017 | Children with DS had a similar caries experience to children without DS | 2 | 1 | 2 |  |  |
| 8 | Lee et al  **Dental caries and salivary immunoglobulin A in Down syndrome children**  Journal of paediatric Child health | 2004 | This study suggests that Down syndrome children have a lower prevalence of dental caries than normal children, which might be due to the higher amounts of S. mutans specific IgA antibodies. In addition, these observations need to be confirmed continuously in other well-designed studies. | 1 | 2 | 1 |  |  |
| 9 | Mathias, M et al  **Some factors associated with dental caries**  **in the primary dentition of children**  **with Down syndrome**  European Journal of Paediatric Dentistry | 2011 | The case group and control group presented a similar caries index, whereas gingivitis was shown to be present from early childhood in children with Down syndrome. | 1 | 2 | 1 |  |  |
| 10 | Stabholz et al    **Caries experience, periodontal treatment needs, salivary PH, and Streptococcus mutans counts in preadolescent Down syndrome population**  Special Care in Dentistry | 1991 | In the DS group, 25% of DMFS is 'F', compared with 56% of healthy group. MR group is only 12%. 72% of healthy group needs treatment with restorations +/- extractions. DS = 46% and MR = 33%. Authors hypothesise that because DS and MR (institutionalised) is more expensive, more complex and requires more specialised personnel, only a small proportion of their needs are met. They also hypothesise that the low caries rate in DS is due to late eruption of teeth and spaced dentition. | 1 | 2 | 2 |  |  |
| 11 | Subramanium, P et al  **Assessment of salivary total antioxidant levels and oral health status in children with Down syndrome**  Special Care Dentistry | 2014 | Dental caries in primary dentition was higher in both DS and normal children.  2. Oral hygiene of children with DS was significantly poorer than that of normal children.  3. TAC of saliva was significantly higher in normal children than DS children.  4. In children with DS, TAC of saliva showed an inverse relation with dental caries, which was significant in the primary dentition.  5. Levels of SA in saliva were significantly higher in DS children than normal children. | 2 | 2 | 2 |  |  |
| **Appendix 3B. Findings and Quality Appraisal: Autism** | | | | | | | | |
| **Study ID** | **Authors/ Title/ Citation** | **Year** | **Study Author Conclusions** | **Risk of Bias (RoB) Assessment (Newcastle-Ottawa Scale)** | | | | **Review Author Additional Notes** |
|  |  |  |  | **Selection**  **(maximum 5 points)** | **Comparability**  **(maximum 2 points)** | **Outcome**  **(maximum 3 points)** | **RoB** |  |
| 12 | Al-Maweri et al  **Oral lesions and dental status of autistic childfren in Yemen: A case-control study**  Journal of International Society of Preventive & Community Dentistry | 2014 | Children with autism in Yemen have a significantly higher prevalence of caries, gingivitis and poor OH. However, children with autism have a higher RI and MNI than children without autism | 2 | 1 | 3 |  |  |
| 13 | Bhandary et al  **Salivary biomarker levels and oral health status of children with autistic spectrum disorders: a comparative study**  European Archives of Paediatric Dentistry | 2017 | Salivary pH, salivary buffering capacity lower in ASD children, caries prevalence higher in ASD children when compared to their healthy siblings | 2 | 2 | 1 |  |  |
| 14 | Du et al  **"Oral health among preschool children with autism spectrum disorders:**  **A case-control study"**  Autism | 2014 | In Hong Kong, differences in oral health status exist among pre- school children with and without ASDs. Children with ASD tend to have less caries experiences and better oral hygiene and gingival health than children without ASD. Similar experiences of tooth wear, trauma, malocclusion and oral mucosal lesions were observed in children with and without ASD. Nonetheless, the prevalence and extent of oral health problems is considerable and warrants special attention given the challenges of caring for children with ASD in the dental setting | 3 | 2 | 1 |  |  |
| 15 | El Khatib et al  **Oral health status and behaviours of children with Autism Spectrum Disorder: a case-control study**  International Journal of Paediatric Dentistry | 2014 | ASD children largely behave negatively for examination 2) ASD children have poorer oral hygiene and gingivitis, more likely to have tooth brushing problems 3) caries prevalence similar between groups. In primary dentition, ASD children more untreated caries. In mixed stage, ASD children less filled teeth than healthy kids 4)ASD children, fewer of them had access to dental care | 2 | 2 | 3 |  |  |
| 16 | Fakroon et al  **Dental caries experience and periodontal treatment needs of children with autistic spectrum disorder**  European Archives of Paediatric Dentistry | 2014 | Libyan children with ASD are more likely to have lower DMFT scores and higher periodontal treatment needs than healthy controls. Further efforts are required to develop preventive dental programmes targeting children with ASD as well as their families and carers and to meet the treatment needs for such special need group. | 5 | 2 | 2 |  |  |
| 17 | Jaber et al  **Dental caries experience, oral health status and treatment needs of dental patients with autism**  Journal of Applied Oral Science | 2011 | Children with autism had higher caries prevalence and unmet need compared with non- Autistic children | 1 | 2 | 1 |  | For children with any caries, they are 2.5 x more likely to have a DMFT > 0 (2.4 vs 0.9) on a whole group basis, the Autistic children are 5 x as likely to have a DMFT > 0 (1.9 vs 0.4) and Autistic children receive 60% less treatment. |
| 18 | Namal et al  **Do autistic children have higher levels of caries? A cross sectional study in Turkish children**  Journal of Indian Society of Pedodontics and Preventive Dentistry | 2007 | ASD not a risk factor for caries. ASD children have a lower caries experience compared with non-ASD children (p<0.05) yet have more missing teeth than non-ASD group and are more likely to receive extraction rather than restoration | 2 | 1 | 1 |  |  |
| 19 | Yoshoda et al  **Oral health status and parental perception of child oral health related quality-of-life of children with autism in Bangalore, India**  Journal of Indian Society of Pedodontics and Preventive Dentistry | 2014 | Poor OH may have a negative impact on OHRQoL of children with Autism - higher caries prevalence | 0 | 1 | 3 |  |  |
| **Appendix 3C. Findings and Quality Appraisal: Mixed or Non-Specific Learning Disabilities** | | | | | | | | |
| **Study ID** | **Authors/ Title/ Citation** | **Year** | **Study Author Conclusions** | **Risk of Bias (RoB) Assessment (Newcastle-Ottawa Scale)** | | | | **Review Author Additional Notes** |
|  |  |  |  | **Selection**  **(maximum 5 points)** | **Comparability**  **(maximum 2 points)** | **Outcome**  **(maximum 3 points)** | **RoB** |  |
| 20 | Bakry et al  **Risk factors associated with caries experience in children and adolescents with intellectual disabilities**  The Journal of Clinical Pediatric Dentistry | 2012 | Caries experience in ID children is lower in the primary dentition but higher in the permanent dentition compared to children without ID. The severity of an ID, and the extent to which a child with ID depends on others for care correlates with an increased caries risk. | 3 | 2 | 1 |  |  |
| 21 | Forsberg et al  **Dental health and dental care in severely mentally retarded children**  Swedish Dental Journal | 1985 | "The prevalence of caries among the SMR-children was generally lower than among the healthy children" "The SMR-children had been offered dental care to the same extent as healthy children" | 2 | 1 | 1 |  |  |
| 22 | Moreira et al  **Does intellectual disability affect the development of dental caries in patients with cerebral palsy?**  Research in Developmental Disabilities | 2012 | CP children with intellectual disability had significantly more carious lesions than those children with CP without a learning disability, and those children with no CP or learning disability. This means that in this study, learning disability presence was a significant contributing factor to the presence and development of carious lesions. | 2 | 1 | 1 |  |  |
| 23 | Jokic et al  **Dental caries in disabled children**  Collegium Antropologicum | 2007 | No statistically significant difference in caries prevalence in deciduous, mixed or permanent dentitions between groups. | 0 | 1 | 1 |  | Authors state no difference in caries prevalence, but there'd appear to be higher caries rates (dft and DMFT) in the disabled population compared to the disabled population. |
| 24 | Pope et al  **The dental status of cerebral palsied children**  Pediatric dentistry | 1991 | Caries levels similar but children with CP, where treatment is delivered are more likely to have extractions performed and less likely to have teeth restored. | 2 | 2 | 1 |  | Six years old and under, mentally handicapped children with CP had fewer sound teeth compared with non-mentally handicapped CP children (numbers not given). In 6-11 year-olds, there was no statistical difference in sound teeth numbers between the CP children with and without LD. |
| 25 | Palin et al  **Dental health of 9-10-year-old mentally retarded children in Eastern Finland**  Community Dentistry and oral Epidemiology | 1981 | In comparison with the healthy, the retarded children are not given enough dental care with respect to their treatment need. The children at home and included in the special welfare had the best dental health among the mentally retarded. The children in institutions, even though in regular care, had poor dental health. In all respects the situation in the mildly or moderately retarded appeared the poorest among retarded children. The present findings evidently emphasize the need of counseling for oral health habits among those responsible for the retarded children, and the need for effective preventive measures in mentally retarded children, whether at home or in an institution. | 4 | 2 | 2 |  |  |

* Quality assessment: S: Selection (maximum 5 points); C: Comparability (maximum 2 points); O: Outcome (maximum 3 points); RoB: Risk of Bias

Low risk: high risk; unclear risk;
